# Emerging Strategies to Combat ESKAPE Pathogens in the Era of Antimicrobial Resistance: A Review

**DOI:** 10.3389/fmicb.2019.00539

**Published:** 2019-04-01

**Authors:** Mansura S. Mulani, Ekta E. Kamble, Shital N. Kumkar, Madhumita S. Tawre, Karishma R. Pardesi

**Affiliations:** Department of Microbiology, Savitribai Phule Pune University, Pune, India

**Keywords:** ESKAPE, multidrug resistance, alternative therapy, combination therapy, phage therapy, antimicrobial peptides, silver nanoparticles, photodynamic light therapy

## Abstract

The acronym ESKAPE includes six nosocomial pathogens that exhibit multidrug resistance and virulence: *Enterococcus faecium, Staphylococcus aureus, Klebsiella pneumoniae, Acinetobacter baumannii, Pseudomonas aeruginosa*, and *Enterobacter* spp. Persistent use of antibiotics has provoked the emergence of multidrug resistant (MDR) and extensively drug resistant (XDR) bacteria, which render even the most effective drugs ineffective. Extended spectrum β-lactamase (ESBL) and carbapenemase producing Gram negative bacteria have emerged as an important therapeutic challenge. Development of novel therapeutics to treat drug resistant infections, especially those caused by ESKAPE pathogens is the need of the hour. Alternative therapies such as use of antibiotics in combination or with adjuvants, bacteriophages, antimicrobial peptides, nanoparticles, and photodynamic light therapy are widely reported. Many reviews published till date describe these therapies with respect to the various agents used, their dosage details and mechanism of action against MDR pathogens but very few have focused specifically on ESKAPE. The objective of this review is to describe the alternative therapies reported to treat ESKAPE infections, their advantages and limitations, potential application *in vivo*, and status in clinical trials. The review further highlights the importance of a combinatorial approach, wherein two or more therapies are used in combination in order to overcome their individual limitations, additional studies on which are warranted, before translating them into clinical practice. These advances could possibly give an alternate solution or extend the lifetime of current antimicrobials.

## Introduction

Wonder drug penicillin started the era of antibiotics in 1928 and since then it has tremendously developed modern medicine. Persistent use of antibiotics, self-medication, and exposure to infections in hospitals has provoked the emergence of multidrug resistant (MDR) bacteria responsible for 15.5% Hospital Acquired Infection (HAIs) in the world (Rice, 2008; Allegranzi et al., 2011; Ibrahim et al., 2012; Pendleton et al., 2013). The term “ESKAPE” encompasses six such pathogens with growing multidrug resistance and virulence: *Enterococcus faecium, Staphylococcus aureus, Klebsiella pneumoniae, Acinetobacter baumannii, Pseudomonas aeruginosa*, and *Enterobacter* spp. (Rice, 2008). ESKAPE pathogens are responsible for majority of nosocomial infections and are capable of “escaping” the biocidal action of antimicrobial agents (Rice, 2008; Navidinia, 2016).

A systematic review of clinical and economic impact of antibiotic resistance reveals that, ESKAPE pathogens are associated with the highest risk of mortality thereby resulting in increased health care costs (Founou et al., 2017). World Health Organization (WHO) has also recently listed ESKAPE pathogens in the list of 12 bacteria against which new antibiotics are urgently needed (Tacconelli et al., 2018). They describe three categories of pathogens namely critical, high and medium priority, according to the urgency of need for new antibiotics. Carbapenem resistant *A. baumannii* and *P. aeruginosa* along with extended spectrum β-lactamase (ESBL) or carbapenem resistant *K. pneumoniae* and *Enterobacter* spp. are listed in the critical priority list of pathogens; whereas, vancomycin resistant *E. faecium (VRE)* and methicillin and vancomycin resistant *S. aureus* (MRSA and VRSA) are in the list of high priority group. The mechanisms of multidrug resistance exhibited by ESKAPE are broadly grouped into three categories namely, drug inactivation commonly by an irreversible cleavage catalyzed by an enzyme, modification of the target site where the antibiotic may bind, reduced accumulation of drug either due to reduced permeability or by increased efflux of the drug (Santajit and Indrawattana, 2016). They are also able to form biofilms that physically prevent the immune response cells of host as well as antibiotics to inhibit the pathogen. Moreover, biofilms protect specialized dormant cells called persister cells that are tolerant to antibiotics which cause difficult-to-treat recalcitrant infections (Lewis, 2007).

The general antimicrobial therapy to effectively treat infections involves the use of antibiotics either singly or in combination. With every passing year, the overall number of antibiotics effective against ESKAPE is declining, which is predisposing us toward a future with antibiotics that are ineffective. Analysis of the antibiotic lists recommended in the Clinical & Laboratory Standards Institute (CLSI) guidelines revealed that many antibiotics suggested against ESKAPE since 2010 have been deleted with addition of relatively few antibiotics/antibiotic combinations. Furthermore, there are incidences of resistance reported against some of these newly added antibiotics (Table 1). It is, therefore, imperative to find alternative ways to treat infections especially those caused by ESKAPE pathogens.

**Table 1:** Antibiotics added/revised and eliminated against ESKAPE from CLSI document M100 since 2010.

**Antibiotics added/with revised breakpoint**	**Tested against pathogen**					
	**Ef**	**S**	**K**	**A**	**P**	**E**
Amikacin, Amoxicillin-clavulanate, Ampicillin-sulbactam, Cefaclor, Cefamandole, Cefdinir, Cefmetazole, Cefonicid, Cefotetan, Cefpodoxime, Cefprozil, Cefuroxime, Kanamycin, Loracarbef, Netilmicin, Oxacillin, Tobramycin		0				
Aztreonam			1			1
Cefazolin, Cefepime, Ceftazidime		0	1			1
Cefoperazone, Moxalactam		0			0	
Cefotaxime, Ceftizoxime, Ceftriaxone		0	1		0	1
Ceftaroline		1	1			1
Ceftazidime-avibactam, Ceftolozane-tazobactam			1			1
Cephalothin		0	0			0
Colistin, Piperacillin					1	
Dalbavancin, Telavancin, Oritavancin, Tedizolid	1	1				
Doripenem, Imipenem, Meropenem		0	1	1	1	1
Ertapenem		0	1	1		1
Mezlocillin				0		
Nalidixic acid			0			0
Piperacillin-tazobactam, Ticarcillin-clavulanate		0			1	
Ticarcillin			0	0	0	0

*Please note that the table includes ONLY those antibiotics which are deleted or newly added since 2010*.

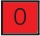
 *= Antibiotics deleted from CLSI guidelines between 2010 and 2018; 1, New antibiotics added in the CLSI guidelines since 2010*; 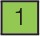 *= No resistance reported till date*; 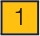 *= Resistance reported; Ef, E. faecium; S, S. aureus; K, K. pneumoniae; A, A. baumannii; P, P. aeruginosa; E= Enterobacter spp*.

*S = (Long et al., 2014; Chan et al., 2015; Nigo et al., 2017); K = (Zowawi et al., 2015; Vuotto et al., 2017; Stanley et al., 2018); A = (Göttig et al., 2014; Goic-Barisic et al., 2017; Nowak et al., 2017; Caio et al., 2018; Chuang and Ratnayake, 2018); P = (Prakash et al., 2014; Gill et al., 2016; Alipour et al., 2017; Mohapatra et al., 2018; Palavutitotai et al., 2018)*;

*E = (Lee et al., 2015; Babouee Flury et al., 2016; Zeng et al., 2016; Kulengowski et al., 2018)*.

**Figure 1:**
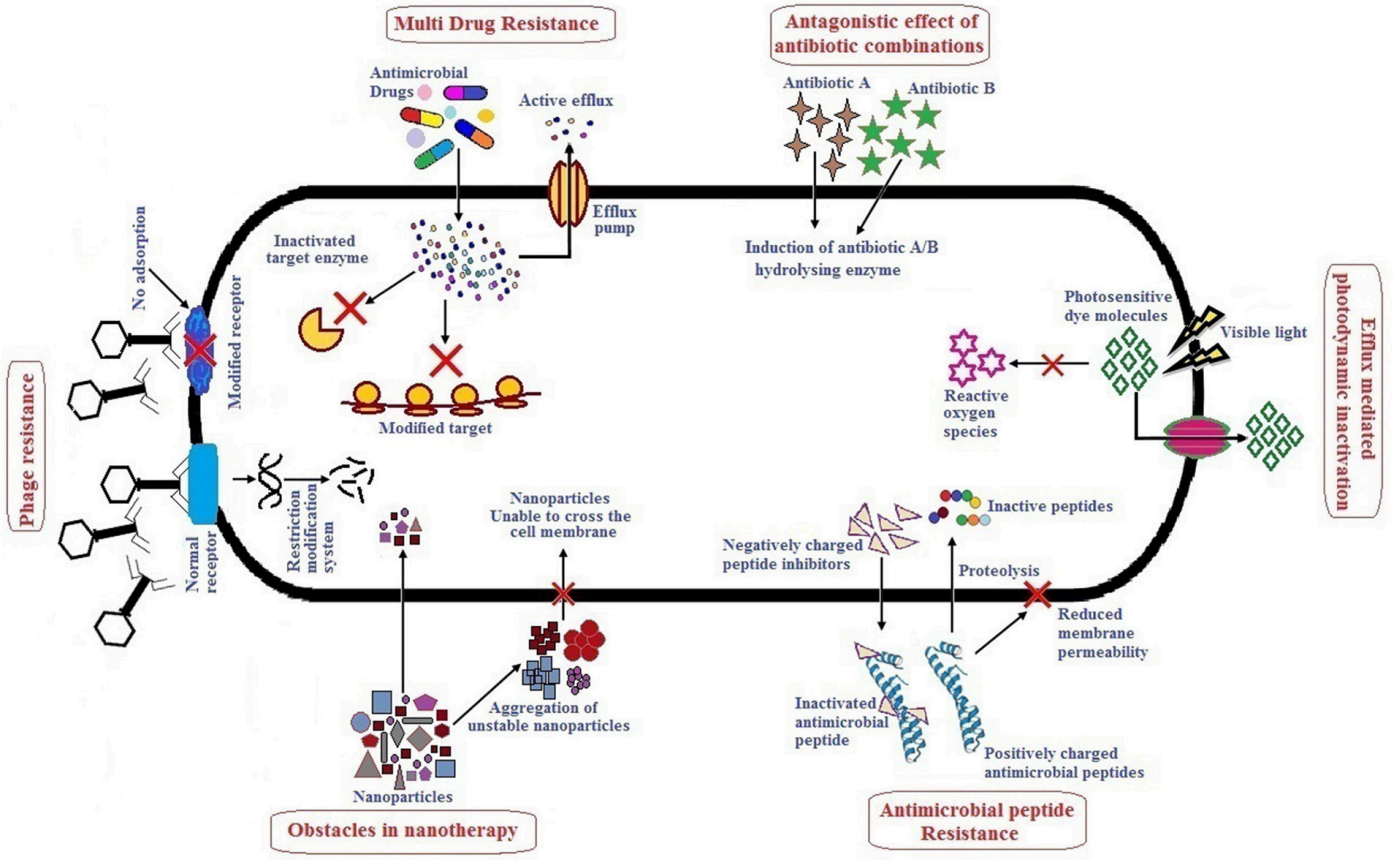
Major challenges of antibacterial monotherapies.

Alternative therapies that are currently in practice or under trial include the use of antibiotics in combination or with adjuvants, bacteriophage therapy, use of antimicrobial peptides, photodynamic therapy, antibacterial antibodies, phytochemicals and nanoparticles as antibacterial agents (Mandal et al., 2014; Kaur, 2016). There are several reviews published which describe alternative/novel therapies against MDR pathogens, however, not many have specifically focused on the ESKAPE group as a whole. These reviews describe the various therapeutic agents used with respect to their dosage, mode of action, pharmacokinetics and pharmacodynamics, stability, and toxicity. Although many alternative therapies reported have shown promising results *in vitro*, their efficacy when applied *in vivo* may not be the same due to one or several limitations. It therefore, becomes necessary to understand the action of these therapeutic agents *in vivo*. This review highlights therapies used to treat ESKAPE infections namely, antibiotics in combination or with adjuvants, bacteriophage therapy, antimicrobial peptides, photodynamic light therapy, and nanoparticles which have received significant attention in the last 5 years. Hereby, we have also tried to include studies especially focusing on *in vivo* efficacy of above mentioned therapy/therapeutic agents, their advantages and limitations. Figure 1 summarizes the major limitations of each alternative therapy which has been elaborately discussed in the following sections. To improve the efficiency and/or minimize the limitations of any therapy/therapeutic agents, combinatorial approaches are suggested. It involves use of two or more monotherapies together (co-administered, functionalized, or conjugated) which is also further described. Altogether, combinatorial approach could be a prospect for exploring novel alternate solutions against ESKAPE.

## Antibiotics in Combination

Antibiotics in combination have been tested as a treatment method by a number of researchers because the possibility of a pathogen to develop resistance against a combination of two drugs is much less than that against a single drug. Similarly, the synergistic effect of combined antibiotics is more than that of the individual antibiotic. A combination of drugs also increases the spectrum of coverage (Vazquez-grande and Kumar, 2015) and has been found to be beneficial in severe infections caused by multiple pathogens (Ahmed et al., 2014). Some of these combinations tested against the ESKAPE are listed in Table 2. The problem of antibiotic resistance is so severe that it has become necessary to try combinations of the most recently synthesized antibiotics and/or last resort antibiotics to study their potential in antimicrobial therapy. The Gram positive members of the ESKAPE, *E. faecium* and *S. aureus*, have been tested against a combination of fosfomycin and daptomycin which has shown to successfully clear infection (Hall Snyder et al., 2016; Coronado-Álvarez et al., 2018). The former is a broad spectrum antibiotic that has shown promising results against Gram negative bacteria while the latter is a last resort antibiotic used to treat infections caused by *E. faecium* and *S. aureus*. Despite being a resistant pathogen there is a lack of substantial research in antibiotic combination therapy against *E. faecium* over the last 5 years. Most combinations tested against *S. aureus in vitro* include daptomycin or vancomycin with other antibiotics including ceftaroline, a newly added antibiotic to the CLSI guidelines. The effect of these and other such combinations have also been tested in various mouse models which cleared away the *S. aureus* infection with minimal to no toxicity. Colistin (polymyxin E) is the last resort antibiotic prescribed against Gram negative bacilli. In recent years, research has been conducted to treat infections caused by *K. pneumoniae* and *A. baumannii* using a combination of colistin or tigecycline with other antibiotics and has shown promising results *in vitro* and in cohort studies.

**Table 2:** Alternative strategies against ESKAPE pathogens.

**Target ESKAPE**	**Model used**	**Agent used and dosage details**	**Route of administration**	***In vivo* efficacy: log reduction of pathogen; % survival of host**	**Other advantages**	**References**
**ANTIBIOTICS IN COMBINATION**						
Ef	*Galleria mellonella* larvae infection model	Rifampin (0.5–2) μg/ml+Tigecycline (0.03 μg/ml)/Vancomycin (64, 128 μg/ml)/Linezolid (2 μg/ml)	Injection into hemocoel	20–73% survival	–	Skinner et al., 2017
S	Retrospective study 2011–2017	Fosfomycin+Daptomycin/Oxacillin/Vancomycin	–	Successful treatment in 81% patients	–	Coronado-Álvarez et al., 2018
S	Wistar mouse	Rifampin (20 mg/kg) + Flucloxacillin (200 mg/kg)/ Moxifloxacin (10 mg/mg)	Intraperitoneal (IP)	4 log decrease with both combinations	Antibiofilm	Greimel et al., 2017
S	Wistar rats	Gentamicin (8, 50 mg/kg) + 4- (Benzylamino) cyclohexyl 2-hydroxycinnamate (16, 64 mg/kg)	–	4 log reduction; 100% survival	Antibiofilm	Balamurugan et al., 2015
K	Murine complicated urinary tract infection model	Meropenem (400 mg/kg) + Nacubactam (150 mg/kg)	–	>3 log reduction in isolates resistant to meropenem-nacubactam	–	Monogue et al., 2018a
A	Murine Thigh and Lung Infection Models	Colistin + Tazobactam/Avibactam	IP	No effect	–	Monogue et al., 2018b
P	Murine thigh infection model	Imipenem (4 or 5 g/day with a 1-g loading dose)+ Tobramycin (7 mg/kg)	–	≥2.5 or ≥1.50 log reduction	–	Yadav et al., 2017
P	Murine infection model	Colistin (2.5–5 mg/kg) + Rifampicin (10 mg/kg)	Subcutaneous	~5 log reduction	–	Cai et al., 2018
E	*G.mellonella* infection model	Colistin (2.5 mg/kg) + Imipenem (15 mg/kg)	Injection into hemocoel	80–90% survival	–	Yang et al., 2018a
**PHAGE THERAPY**						
Ef	Murine bacteraemia model	Phage ENB6 and C3 (A2 morphotype group), of 3 × 10^8^ PFU	IP	Single dose: 50% survival; Multiple doses: 100% survival	Immunocompatible	Biswas et al., 2002
S	65-year-old woman with Corneal abscess	SATA-8505 (ATCC PTA-9476)	Topical (eye drops and nasal spray) and intravenous (IV)	Eradication of pathogen and stabilization of ocular signs	-	Fadlallah et al., 2015
S	Case series (six human subjects suffering from diabetic foot ulcer)	Commercial staphylococcal phage Sb-1; (0.05–0.4 ml of 10^7^-10^8^ PFU/ml)	Topical	–	Wound healing within 7 weeks	Fish et al., 2016
S	Rabbit osteomyelitis model	Cocktail of phages (SA-BHU1, 2, 8, 15, 21, 37 and 47); 15 μl of 5 × 10^12^ PFU/ml of each	Intramuscular (IM)	100% survival	Viruses with stable genomic structure	Kishor et al., 2016
S	Murine bacteraemia model	Phage SLPW Podoviridae; 0.2 mL of 1 × 10^9^ PFU	IP	~100% decrease in bacterial count; up to 80% survival	Stable up to 45°C, pH 6- 10; Immunocompatible	Wang et al., 2016
S	Murine wound infection model	MR 5 and MR 10, Myoviridae; free phage 10^9^ PFU/50 μl and its liposomal formulation	Topical	Up to 3 log reduction on day 10	Wound healing within 7 days	Chhibber et al., 2018
K	Murine burn wound infection model	Five phages KØ1 to 5 (10^7^ PFU/ml) in equal proportion as a cocktail or loaded in liposomes	IP	>4 log reduction; 100% survival	Enhanced wound healing	Chadha et al., 2017
A	Case study 77-year-old man with post-operative infection	Five phages active against 104 *A. baumannii* from the NMRC's phage-Biolog system 2.14 × 10^7^ PFU/mL	Intravenous (IV)	No effect	–	LaVergne et al., 2018
A	Murine wound infection model	Cocktail of AB-Army 1 and AB-Navy 1 to 4 (~4 × 10^9^ PFU)	Topical, IP or loaded on TegadermTM bandage	3 log tested *in vitro*; ND	Antibiofilm; Reduction in wound bio-burden and size (loss of capsule production in phage infected *Acinetobacter*)	Regeimbal et al., 2016
A	Murine wound infection model	Acinetobacter phage- Siphoviridae; (400 μl of 3 × 10^9^ PFU/ml)	Topical	100% clearance of infection in 8 days	Reduction in wound bio-burden but no difference in time required for wound healing	Shivaswamy et al., 2015
A	*G.mellonella* infection model	Phage WCHABP1 and WCHABP12 Myoviridae; 10^4^ PFU/larva	Injection into hemocoel	75% survival	No integrase and repressor genes were identified in both bacteriophages	Zhou et al., 2018
A	Case study (68 year old patient with infected diabetic patient)	Cocktails of AB Phage ΦPC (AB-Navy1, 4, 71, 97, and AbTP3Φ1); ~10^9^ PFU/dose	Percutaneous catheter, IV	Clearance of infection	–	Schooley et al., 2017
P	Murine infected model	Phage PEV20, Podoviridae; Inhalable powder 2 × 10^7^ PFU/mg	Intranasal and intratracheal	5 log reduction	Non-toxic	Chang et al., 2018
P	Case study (2-year-old boy with a history of DiGeorge syndrome)	Cocktail from US Navy library of bacteriophages 3.5 × 10^5^ every 6 h. six doses total and resumed after 11 days	IV	Blood cultures negative After phage treatment	-	Duplessis et al., 2018
P	Case study (61 year old male patient with acute septicaemia and large necrotic pressure sores)	Phage BFC1 50 μl IV infusion every 6 h for 10 days; Wounds irrigation- 50 ml BFC1 every 8 h for 10 days	IV infusion and Topical	Clearance of septicaemia immediately after therapy; pressure sores remained infected with several bacterial species, including *P. aeruginosa*	No adverse effects	Jennes et al., 2017
P	*G.mellonella* infection model	Phage KTN4; MOI100	Injection into hemocoel	4–7 log reduction; 100% survival	Antibiofilm; Stable up to 40–70°C	Danis-Wlodarczyk et al., 2016
E & P	Case series (9 UTI patients)	Pyophage	Pyophage instillation by suprapubic catheter	bacterial titers decreased after bacteriophage treatment in six out of nine patients (67%).	No bacteriophage-associated adverse events detected	Ujmajuridze et al., 2018
S, A, & P	Murine sepsis model	Phage against *A. baumanni, P. aeruginosa*, and *S. aureus*	IP	100% reduction in A and P; 100% survival	–	Soothill, 1992; Lin et al., 2017
K & E	*G. mellonella* infection model	Cocktail of Escherichia phage ECP311, Klebsiella phage KPP235, and Enterobacter phage ELP140	–	100% reduction after 5 doses; 90% survival	–	Manohar et al., 2018
**SILVER NANOPARTICLES**						
*S*	Infected *Daniorerio*-Zebrafish model	Sunlight mediated AgNP synthesized using plantextract (HWP AgNP) (10–40 nm)	–	>1 log reduction	Antibiofilm; Non-toxic	Lotha et al., 2018
*S*	Murine wound infection model	Chitosan-coated AgNPs (10–30 nm) (50 μg/g·bw)	Topical	–	Wound healing within 14 days; Non-toxic	Peng et al., 2017
*S*	Rabbit flexor tendon rupture model	Core-shell nanofibrous membranes with embedded AgNPs in PEG/poly (caprolactone) shell and hyaluronic acid/ibuprofen in the core	AgNP Coated implant	–	Antibacterial activity; Prevention of peritendinous adhesion after tendon surgery; Non-toxic	Shalumon et al., 2018
*S*	Murine model	Wound dressing containing nanosilver nanohydrogels (nSnH) along with *Aloe vera*	Topical	–	Antibacterial; Wound healing within 16 days; Non-toxic	Anjum et al., 2016
*S*	Murine wound infection model	Sodium carboxymethylcellulose hydrogel loaded with PEG-coated-AgNPs (19.2 ± 3.6 nm) (500 μg AgNPs/g of hydrogel)	Topical	97.30% reduction; 100% survival	Wound healing within 10 days	Mekkawy et al., 2017
*S*	Murine model	Mesoporous silica nanoparticles coated with AgNPs (Ag-MSNs)	Topical	–	Antibacterial; Wound healing within 5 weeks; Anti-inflammatory	Dong et al., 2016
*S*	Rabbit flexor tendon model	AgNPs embedded in electrospun hyaluronic acid (HA)/polycaprolactone (PCL) nanofibrous membranes (NFMs)	AgNP Coated implant	–	Antibacterial; Prevention of peritendinous adhesion after tendon surgery	Chen et al., 2015
*S & P*	Murine wound infection model	AgNPs (20 nm)/chitosan composite dressing (0.5 mM)	Topical	–	Wound healing within 8 days; Non-toxic	Liang et al., 2016
*S & P*	*G. mellonella* model	Electrochemically synthesized AgNPs (8–10 nm) (6.8 and 3.4 mg/mL)	Injected into hemocoel	–	Antibiofilm; Non-toxic	Pompilio et al., 2018
*S & P*	Murine model	Bilayer composite of Chitosan-AgNPs (5–50 nm) (CS-AgG) on CSGB (*Bletilla striata* polysaccharide) both linked with genipin	Topical	–	Antibacterial; Wound healing; Non toxic	Ding et al., 2017
*S & P*	Murine model	NanoAg (< 50 nm) wound dressings; 100 μL of 1 mg/mL	Topical	–	Antibacterial; Antibiofilm; Biocompatible	Radulescu et al., 2016
*S & P*	Murine model	Collagen nanofiber mats containing AgNPs (25–55 nm)	Topical	–	Antibacterial; Wound healing within 14 weeks; No hypersensitive; Anti-inflammatory	Rath et al., 2016
*S & P*	Gottingen minipigs femur model	Nanocomposite coating formed by polysaccharide 1-deoxylactit-1-yl chitosan (Chitlac) and AgNPs on methacrylate thermosets	AgNP Coated implant	–	Antibiofilm; Anti-inflammatory; Non-toxic	Marsich et al., 2013
**ANTIMICROBIAL PEPTIDE (AMP)**						
*A*	Murine infection model	Chex1-Arg20 amide; 2 and 5 mg/kg	IM	>2 log reduction; >50% survival	Anti-inflammatory	Ostorhazi et al., 2018
*A*	Murine wound infection model	K11, a hybrid peptide of melittin, cecropin A1, and magainin 2	Topical	~100% pathogen clearance; 100% survival	Wound healing (21 days)	Rishi et al., 2018
*P*	Murine infection model	Feleucin-K3 analogs; 5 mg/kg	IP	~ 1 log reduction	Stable; Low toxicity; Antibiofilm	Xie et al., 2018
*S*	*G. mellonella* infection model	PT-13, Plant derived crude extract; 64 μM	Injection into hemocoel	60% survival	–	Al Akeel et al., 2018
*P*	*G. mellonella* infection model	SP-E, a proline-rich pig saliva derived peptide; 6.1 μmol/kg	Injection into hemocoel	~50% survival	Non-toxic	Ciociola et al., 2018
*S & A*	*S. aureus* murine sepsis model, *A. baumannii* infected murine lung model	D-150–177C, peptide modified by attaching C-terminal Cysteine; 5 and 10 mg/kg	IP	70% survival	Non-toxic	Chen et al., 2018
*S & A*	Murine skin infection model	SAAP-148, a LL-37 human cathelicidin inspired peptide used to prepare ointment; 0.125 to 0.5% (w/w)	Topical	87% and complete clearance of *S. aureus* and *A. baumannii*, respectively After a single treatment with 2% (w/w) SAAP-148 ointment	Antibiofilm; Kills persister cells; Reducing bioburden and wound healing; Non-toxic	de Breij et al., 2018
*P*	Murine lung infection model	Esc (1–21)-1c, frog skin derived AMP; 0.1 mg/kg	Intra-tracheal injection	2 log reduction	Anti-inflammatory	Chen et al., 2017
**ANTIMICROBIAL LIGHT THERAPY**						
S	Murine burn infection model	Laser light-50 J/cm^2^; PS-PS- sinoporphyrin sodium (DVDMS)- 2, 5, and 10 μM, 75 min	Topical	4 log reduction at 5 μM of DVDMS with 50 J/cm^2^ light	DVDMS promoted wound healing after burn infections	Mai et al., 2017
S	Murine ulcer infection model	Laser light-LED 410 nm, 50 J/cm^2^; PS-5-ALA; 200 mg/kg	Topical	2 log reduction	–	Morimoto et al., 2014
S	Murine infection model	Light: 660 nm- 45 J/cm^2^- 10 min exposure; household light- 7.5 J/cm^2^- 10 min exposure; PS pentalysine-β-carbonylphthalocyanine Zinc (ZnPc(Lys)5)- 5 μl at 1 mM	Topical	44.3% clearance of infection at 7.5 J/cm^2^	Improved wound healing	Ullah et al., 2018
A	Murine burn infection model	Light- LED 415 nm 72–360 J/cm^2^	Topical	3 log reduction	Antibiofilm	Wang et al., 2017
P	Murine skin abrasion model	Light- 415 nm, 48 J/cm^2^	Topical	5 log reduction	–	Amin et al., 2016

*Ef, Enterococcus faecium; S, Staphylococcus aureus; K, Klebsiella pneumonia; A, Acinetobacter baumannii; P, Pseudomonas aeruginosa; and E, Enterobacter spp*.

Some molecules when combined with antibiotics make an ineffective drug effective. These molecules, named “adjuvants” or “resistance breakers,” have little to no antimicrobial activity of their own (González-Bello, 2017) but inhibit the mechanism of resistance by increasing the uptake of the antibiotic through the bacterial membrane, blocking of efflux pumps, and changing the physiology of resistant cells (i.e., dispersal of biofilms to planktonic cells) (Kalan and Wright, 2011; Bernal et al., 2013). Essential oils and phenothiazines enhance the antimicrobial activity of drugs and also inhibit the transmission of resistance to other populations (Bueno, 2016).

The most popularly known adjuvants are β-lactamase inhibitors while the most recent adjuvant tested to restore meropenem activity is vaborbactam which inhibits *K. pneumoniae* carbapenemase activity (Jorgensen and Rybak, 2018). It has also undergone a clinical trial; registered on ClinicalTrials.gov under identifier NCT02020434; which proved the combination to be safe after testing 41 subjects (Rubino et al., 2018). However, it has a limited inhibition activity as it has been unable to do so with class B and class D β-lactamases. Other β-lactamase inhibitors include avibactam, nacubactam, and tazobactam (Monogue et al., 2018a,b). Metal chelators like EDTA, deferasirox, and deferoxamine also inhibit β-lactamases as these enzymes require metal ions for their activity (Aoki et al., 2010; Santos et al., 2012; Yoshizumi et al., 2013). These chelators have been tested in combination with antibiotics like imipenem, tobramycin, and vancomycin against *S. aureus, P. aeruginosa*, and *E. coli* in murine models with successful decrease in bacterial load. Quorum quenchers, molecules that inhibit quorum sensing thereby inhibiting biofilm formation, have also shown potential to cure infection in combination with certain antibiotics (Balamurugan et al., 2015; Chatterjee et al., 2016). 1-[(2,4-Dichlorophenethyl)amino]-3-Phenoxypropan-2-ol is so far the most promising antimicrobial agent as it has been reported to kill not only persister cells of *P. aeruginosa* but also non-persister cells of all the other ESKAPE members. It has also been shown to enhance killing of antibiotic resistant strains in both planktonic and biofilm forms. Its combination with a variety of antibiotics is shown to kill all of the ESKAPE making it an ideal candidate as an adjuvant (Defraine et al., 2017).

Even though there is increased activity of antibiotics when used in combination against pathogens *in vitro*, there are limited studies demonstrating the same *in vivo* and some among those have proven disadvantageous. If monotherapy selects for a narrow spectrum of resistance, a combination of two or more antibiotics selects for a broad spectrum of resistance defeating the purpose of combination therapy entirely (Vestergaard et al., 2016). Certain combinations that are meant to treat infections tend to have the opposite effect resulting in far worse damage. One antibiotic can lead to the induction of a resistance mechanism against a second antibiotic administered in combination leading to antagonistic effect (Fallah, 2018). A clinical trial conducted in Italy in which infections caused by XDR *A. baumannii* were treated with a combination of colistin and rifampin showed no improvement in survival rates. In fact, this combination led to increased hepatic toxicity (Durante-Mangoni et al., 2013). A similar study using a combination of colistin, tigecycline and carbapenems against *A. baumannii* showed futile results (López-Cortés et al., 2014). Metal chelators have shown to sequester ions not only from bacterial cells but also from host tissue cells (Yoshizumi et al., 2013).

The most recent alternative to antibiotics or their combination that shows a promising future is antibiotic hybrids which have been defined by Domalaon et al. (2018) as synthetic constructs of two or more pharmacophores belonging to an established agent known to elicit a desired antimicrobial effect. This method provides the advantage of combination therapy through the mono therapy approach, where chances of resistance are curbed, while overcoming the problem of non-complementary pharmacodynamic profiles of the individual antibiotics.

The ESKAPE tend to become resistant to either or both antibiotics used in combination with every passing year due not only to natural selection of resistant strains but also horizontal gene transfer from them to sensitive strains. This warrants testing of still new combinations. The result is a never-ending cycle from which there is no escape. It can therefore be concluded that antibiotics in combination may not always be effective and that there is a need for extensive research of alternative strategies.

## Bacteriophage Therapy

Phages are century old therapeutic agents that were used for the treatment of bacterial infections. The discovery of antibiotics was an influential factor in side-lining this ambition (Mann, 2005). The focus on phage therapy has sharpened ever since antimicrobial resistance has been on a dramatic rise. Lytic phages against ESKAPE pathogens have been isolated from hospital wastewater, making them easily available therapeutic agents (Latz et al., 2016). Bacteriophages used for therapy present many advantages such as high host specificity (prevent damage to normal flora, do not infect the eukaryotic cells), low dosages for treatment, rapid proliferation inside the host bacteria, etc. that make them ideal candidates to treat bacterial infections (Domingo-Calap and Delgado-Martínez, 2018). Unlike antibiotics, the advantage of using phages is that, they develop new infectivity and regain an upper hand over bacteria as they mutate alongside their host (Pirnay et al., 2018).

Several studies carried out *in vitro* have proven phages to be effective as antibacterial agents against biofilm and planktonic cells of ESKAPE (Pallavali et al., 2017; Dvořáčková et al., 2018; Jamal et al., 2019). Table 2 gives information of phage therapy studied in various animal models as well as recent case studies and case reports of patients infected with ESKAPE pathogens. Phage therapy carried out in animal wound infection models have shown reduced mortality and enhanced wound healing. Additional studies carried out *in vivo* have also demonstrated efficacy and safety (non-toxic with reduced inflammatory responses) of phages used in treatment of bacterial infections.

Phage therapy, though promising, comes with some limitations. It can, however, be overcome by appropriate modifications (Wittebole et al., 2014; El-Shibiny and El-Sahhar, 2017; Domingo-Calap and Delgado-Martínez, 2018). High specificity of the phages can be considered as both advantageous and a limiting factor. Monophage therapy involves the need to check the efficacy of the phage by testing it *in vitro* against the disease-causing bacteria before applying it to a patient which can be a difficult process. The use of phage cocktails, comprising of a combination of phages acting against different bacterial species or strains, can avoid these problems (Chan et al., 2013). International experts believe that an ideal phage cocktail should be prepared using phages belonging to different families or groups such as having broad host range, high adsorption ability to the highly conserved cell wall structures in bacteria. Using such phage cocktails may reduce the emergence of phage resistant bacterial population. However, others advocate strategies wherein individual active phages are applied sequentially to the patient. In clinical practice, however, it appears to be difficult (Rohde et al., 2018).

Genomic characterization of phages is very important so as to predict their “safety” in therapeutic applications as demonstrated by several experts in this field. Phages can be vectors for horizontal gene transfer in bacteria, sometimes being involved in exchange of virulence or antibiotic resistance genes making a microbe more pathogenic or resistant to an antibiotic (Chen and Novick, 2009). Phages reported for therapeutic applications should not harbor virulence or antibiotic resistance genes as well as integrases, site-specific recombinases, and repressors of the lytic cycle that may accelerate the transfer/integration of these genes in the host bacterial genome. Algorithms that can be used for predicting lifestyle of a phage, and its virulent traits are available but their database needs to be updated with more genome sequences of phages (Mcnair et al., 2012). Two recent reviews excellently describe the work flow to select ideal phage candidates for therapeutic purposes (Casey et al., 2018; Philipson et al., 2018). Recent studies demonstrating *in vivo* efficacy of phages against ESKAPE infections have used fully characterized phages that show no virulence or antibiotic resistance genes, are considered safe as they do not exhibit any allergic or immune response, and are also reported to remain stable at varied pH and temperature which make them ideal candidates for therapy (Fish et al., 2016; Kishor et al., 2016; Wang et al., 2016; Zhou et al., 2018).

Similarly, it has also been reported that the bacterial strains used for phage production should ideally be free of functional prophages. These prophages may get induced and contaminate the phage preparation. However, a recent report discusses the risk benefit evaluation that needs to be done in highly experimental treatments of patients infected with MDR pathogens such as ESKAPE (Rohde et al., 2018).

Another limitation reported is the stability of phages and their proper administration in order to reach the site of action. Phage formulations are ingested orally, administered nasally or applied topically (Malik et al., 2017; Cooper et al., 2018). Studies have shown improved efficacy of phage when entrapped with liposomes (Singla et al., 2016; Chadha et al., 2017; Malik et al., 2017; Chhibber et al., 2018). Phages can be targeted at the infection site in the form of powdered formulations (Chang et al., 2018). Phage derived product like phage encoded lytic enzymes showing function similar to lysozyme can also be used as an antibacterial agent or can be combined with other antimicrobials like antibiotics to improve efficacy of treatment (Lin et al., 2017). A phage derived protein, “endolysin” is reported for its antibacterial and antibiofilm activity against ESKAPE (Viertel et al., 2014; Gong et al., 2016; Rios et al., 2016; Lin et al., 2017; Zhang et al., 2018). V12CBD a recombinant protein derived from bacteriophage lysine, PlyV12, was able to attenuate virulence of *S. aureus*, and enhance its phagocytosis in mice (Yang et al., 2018b).

Several commercial phage preparations which can be used against ESKAPE are available some of which include, “Stafal,” “Sextaphage,” “PhagoBioDerm,” and “Pyophage”. Stafal (Bohemia Pharmaceuticals, Slovakia) is an antistaphylococcal phage preparation, Sextaphage (Microgen, ImBio Nizhny Novgorod, Russia) is a cocktail against *P. aeruginosa and E. coli* while, Pyophage (Georgian Eliava Institute of Bacteriophage, Microbiology, and Virology) contains bacteriophages specifically eliminating causative agents of pyoinflammatory and enteric diseases. PhagoBioDerm, a polymeric bandage impregnated with cocktail of phages, ciprofloxacin, and other active ingredients ensured for sustained release of phages to treat ulcers or wound infections caused by *S. aureus* and *P. aeruginosa* (Markoishvili et al., 2002). Clinical potential of these preparations has been investigated further to determine their broad spectrum activity against other strains *in vitro, in vivo* efficacy in animal models as well as through several case studies or clinical trials described below.

Recent human case studies involving treatment of ESKAPE associated infections with different phages are described in Tables 2, 3. Readers may refer to previous reviews published on phage therapy which have described in numerous studies (Abedon et al., 2011; Chan et al., 2013; Górski et al., 2017; Lin et al., 2017; Sybesma et al., 2018). Most of the case studies report phage therapy given to patients on a compassionate care basis where antibiotic treatment fails.

**Table 3:** Alternative strategies used in combination against ESKAPE pathogens.

**Target ESKAPE member**	**Study model**	**Agent used and dosage details**	**Efficacy: log reduction of pathogen using combination; % survival of host, additional advantage**	**References**
**PHAGE + ANTIBIOTIC**				
P	Case study (76 year old male with postoperative infection after arch replacement surgery)	Phage OMKO1 titer of 10^7^ PFU/ml + Ceftazidime (0.2 gm/ml)−10 ml of this combination was injected into the mediastinal fistula which was in continuity with the perigraft collection	Eradication of *the P. aeruginosa* infection	Chan et al., 2018
K	*in vitro*–antibiofilm assay, *in vivo*—murine model (for raising antibodies)	KPO1K2, Podoviridae; free and liposome encapsulated (1.2 × 10^13^ in 200 μl, MOI = 1) + amikacin (40 μg/ml)	*in vitro*: 94.6% reduction in intracellular pathogen load; Antibiofilm activity (liposome entrapped phage −7 ± 0.1 logs reduction in bacterial count upto day 4 of biofilm formation)	Singla et al., 2016
P	*in vitro*–antibiofilm assay	NP1 and NP3 phages + ceftazidime, ciprofloxacin, colistin, gentamicin or tobramycin	Synergy between phage and ciprofloxacin/ tobramycin reduced bioburden within the biofilm	Chaudhry et al., 2017
P	*in vitro*	Phage PEV20 (10^10^ PFU/mL; MOI 0.1 and 100) + ciprofloxacin, amikacin, aztreonam, colistin or tobramycin	No obvious growth of pathogen throughout 24 h of incubation for ciprofloxacin or amikacin in combination with phage PEV20	Lin Q. et al., 2018
**SILVER NANOPARTICLES + ANTIBIOTICS**				
*A*	*in vivo* (murine peritonitis model)	Citrate capped AgNPs (5–12 nm, 2 mg/kg) + Polymyxin B (10 μg/kg)	60% survival; Non-toxic; Anti-inflammatory	Wan et al., 2016
S	*in vitro*	AgNPs (synthesized using two actinobacteria, 5–50 and 5–20 nm) + ampicillin, kanamycin /tetracycline	Antibiofilm; Non toxic	Wypij et al., 2018
A	*in vitro*	AgNPs (synthesized using *A. calcoaceticus*, 8–12 nm) + tetracycline, erythromycin or doxycycline	MBEC of combination with AgNPs was 0.5 mg/ml for doxycycline and 4 mg/mL for erythromycin	Singh et al., 2018
S & P	*in vitro*	AgNPs (synthesized using *Garcinia indica* fruit extract, 5 and 30 nm) + tetracycline	Increase in zone of inhibition in S and P with combination	Sangaonkar and Pawar, 2018
P	*in vitro*	Citrate-capped silver nanoparticles (10 nm and 20 nm) + tobramycin	Antibiofilm activity observed using combination	Habash et al., 2017
S, K, & P	*in vitro*	AgNPs (synthesized using Actinomycetes, 59 and 28 nm) + kanamycin,ampicillin or tetracycline	Increase in zone of inhibition using combination	Golińska et al., 2016
**ANTIMICROBIAL PEPTIDES (AMP)** **+** **NPS;**				
Ef	*in vitro*	Synthetic peptide-immobilized gold nanoparticles; 250 μg/ml	Reduced bacterial load (by 0.4 OD units)	Kuo et al., 2016
S	*in vitro*	AMP TP359 covalently linked to silver coated single walled carbon nanotubes	100% reduction	Chaudhari et al., 2016
S & P	*in vitro*	Paenipeptin analog (synthetic linear lipopeptide analogs) + rifampicin, clarithromycin, or erythromycin	Reduction in cell count of biofilms- *S. aureus*−5 logs and *P. aeruginosa*−3 logs	Moon et al., 2017
K	*in vitro*	SPR741, a cationic peptide derived from Polymyxin B + antibiotics	128-fold reduction in MIC with rifampin and clarithromycin –	Corbett et al., 2017
**ANTIMICROBIAL PHOTODYNAMIC LIGHT THERAPY (aPDT)** **+** **ANTIBIOTIC**				
Ef	*in vivo* (G. mellonella infection model)	Light- 660 ± 15 nm, 0.9 J/cm^2^ PS: Methylene blue (MB); Time 30 s + vancomycin-50 mg/kg	100% reduction; ~60% survival	Chibebe Junior et al., 2013
S	*in vitro*	UVC light−254 nm, 6.4 mW + vancomycin (16 μg/mL) or quinupristin/ dalfopristin (32 μg/mL) or linezolid (64 μg/mL)	40% reduction in bacterial load with vancomycin or quinupristin/ dalfopristin and 20% in with linezolid; Synergy reduced viability of cells within the biofilm considerably	El-Azizi and Khardori, 2016
S	*in vitro*	LED light 650 nm, 2.8–22.4 J/cm^2^. PS-MB 6.25–400 μg/mL, ciprofloxacin (0.5 μg/mL)	5 log reduction in bacterial load; 4 log reduction in bacterial cell count in biofilm	Ronqui et al., 2016
**aPDT** **+** **EFFLUX PUMP INHIBITOR**				
S	*in vitro*	Light- 22 J/cm^2^; PS- MB-200 μg/mL + EPI- verapamil 312 μg/mL	3.38 log reduction in biofilm	de Aguiar Coletti et al., 2017
S	*in vitro and in vivo* (Murine wound infection model)	aPDT using NorA EPI-MB hybrid, INF55-(Ac)en-MB	*in vitro*: 6 log reduction; *in vivo*: 100% reduction within 4 days; enhanced wound healing	Rineh et al., 2017
A	*in vitro* and *in vivo* (Murine wound infection model)	*in vitro*: aPDT using NorA EPI-MB hybrid, INF55-(Ac)en-MB	*in vitro*: 5 log reduction; *in vivo*: 100% reduction within 6 days; enhanced wound healing	Rineh et al., 2018
**ANTIBACTERIAL BLUE LIGHT (aBL)** **+** **AgNP**				
S & P	*in vitro, in vivo* (murine burn wound infection model) and Case study (chronic wound infection of horse)	AgNP- chemically synthesized (15–20 nm) + Light-aBL- 460 nm, 2 h exposure after AgNP application	*in vitro*: 10 log reduction in bacterial count in biofilms; *in vivo*: 100% reduction in 2 h in mice; Reduction in wound bioburden; Case study: Wound healed completely within 4 weeks in the horse	Nour El Din et al., 2016
P	*in vitro*	Light- 650 nm, PS: Sodium salt of tetrasulfonated hydroxyaluminum phthalocyanine [Al(OH)Pc(SO3Na)4] + A. pullulans synthesized Au-AgNPs- 14 nm (20 ppm)	3 log reduction	Maliszewska et al., 2018
**BL** **+** **AgNP** **+** **ANTIBIOTICS**				
S	*in vitro*	Light-aBL-460 nm, 250 mW, 1 h, various antibiotics + AgNP- chemically synthesized (15–20 nm). Double combination- AgNP at sub MIC + antibiotics/ aBL; Triple combination- AgNP+ antibiotic+ aBL	100% reduction in 8 h, synergistic activities of AgNP when used in double or triple combinations.	Akram et al., 2016
**aPDT** **+** **AMP**				
S, A, Ef	*in vitro*	MB, MB-PDT at 45 J/cm^2^ or Chlorin 6, Ce6-PDT at 30 J/cm^2^ + AMP- aurein 1.2, AU at 16 μM	*S. aureus*- 6 log reduction, *A. baumannii*- 5 log reduction, *E. faecium*-2.5 log reduction	de Freitas et al., 2018
P	*in vitro*	Rose Bengal (RB)-antimicrobial peptide conjugate to enhance the sonodynamic therapy	7 log reduction, increase in diffusion of RB 2.6 fold through the biofilm	Costley et al., 2017
P	*in vitro*	AMP (CAMEL or pexiganan) aPDT RB at 15–60 J/cm^2^. aPDT with RB and PEX at 5μM each OR RB and CAM at 10μM each	5 log reduction	Nakonieczna et al., 2018
**aPDT** **+** **ADJUVANT**				
P	*in vitro* and *in vivo* (murine wound infection model)	*in vitro*: green light 0, 10, and 20 J/cm^2^; PS- RB- 100 nM; KI- 0–100 mM; *in vivo*: green light-20 J/cm^2^; PS - 500 μM RB alone or mixed with 1M KI	*in vitro*: 7 log reduction; *in vivo*-100% reduction; Inhibition of wound bioburden	Wen et al., 2017
P	*in vitro* and *in vivo* (murine cutaneous ulcer infection model)	*in vitro*: LED 410 nm, 6 and 9 J/cm^2^; PS- 5-Aminolevulinic acid (ALA)-0.05–0.5%, EDTA-2Na 0.001–0.005%; *in vivo*: LED 410 nm, 6 and 9 J/cm^2^; KY jelly with 5-ALA-0.1 and 0.5%, EDTA-2Na 0.001 and 0.005%.	*in vitro*: Combination was less efficient than EDTA-2Na in biofilm inhibition; *in vivo:* 6 log reduction in wound bioburden with 60% healing within 13 days	Katayama et al., 2018
S & A	*in vitro* and *in vivo* (murine skin abrasion model)	*in vitro*: UVA Light 360 ± 20 nm- 0–20 J/ cm^2^-exposure; white light- 400–700 nm, 0–120 J/cm^2^; PS- C60-fullerene (LC16)- 20 μM with or without KI (1 M−30 μl); *in vivo*: UVA Light 360 ± 20 nm- 20 J/cm^2^- exposure; white light- 400–700 nm 120 J/cm^2^; PS- C60-fullerene (LC16)- 200 μM with or without KI (10 mM)	*in vitro*: 1–2 log reduction; *in vivo*: 1–2 log reduction in *A. baumannii* only	Zhang et al., 2015

*Ef, Enterococcus faecium; S, Staphylococcus aureus; K, Klebsiella pneumonia; A, Acinetobacter baumannii; P, Pseudomonas aeruginosa; and E, Enterobacter spp*.

Several case studies have demonstrated the efficacy of phage therapy in treating patients suffering from eye infections (Fadlallah et al., 2015), pancreatitis (Schooley et al., 2017), diabetic foot ulcer (Fish et al., 2016), and urinary tract infection (Ujmajuridze et al., 2018). Fadlallah et al. (2015), reported a case study of a 65 year old woman suffering from a secondary eye infection by VRSA, treated with a well-characterized commercially available lytic phage, SATA-8505 (ATCC PTA-9476) against MRSA. After 6 months of treatment, the patient was diagnosed as negative for cultures of VRSA. This case study suggests that bacteriophage eye-drops can be used as an alternative treatment of infectious keratitis by MDR pathogens. Fish et al. (2016), reported a case involving nine patients suffering from diabetic foot ulcer, who were treated with a preparation of anti-staphylococcal phages (Sb-1) on a compassionate care basis. The average healing time reported was ~7 weeks. However, one ulcer, with poor vascularity required 18 weeks of treatment. Similarly, Ujmajuridze et al. (2018), reported a preclinical pilot study for a double blind RCT registered at ClinicalTrials: NCT03140085 (Leitner et al., 2017). Pyophage were used in this study to treat nine patients suffering from UTI infections. The first phase of this study involved adaptation cycles of the commercial Pyophage preparation to increase its sensitivity toward the uropathogens. In the second phase, six of the nine patients responded to the adapted bacteriophage showing up to five log reduction in CFU of the infecting bacteria. Another study by Schooley et al. (2017) described the case of a 68 year old male diabetic patient having necrotizing pancreatitis which was complicated by an MDR *A. baumannii*. The patient responded to a phage therapy which consisted of a cocktail of nine phages against *A. baumannii*.

Few cases showing partial success of phage therapy in treating the infection are also reported (Jennes et al., 2017; Duplessis et al., 2018). Jennes et al. (2017) reported a case study of 61-year-old man diagnosed with septicaemia caused by a colistin-only-sensitive *P. aeruginosa*. Blood cultures turned negative immediately after BFC1 phage therapy but sores remained infected. No adverse side effects were observed during this therapy. In another study, a 2-year-old boy with a history of DiGeorge syndrome and complex congenital heart disease developed post-operative recalcitrant *P. aeruginosa* bacteraemia. A cocktail of two phages were used to target the infecting pathogen turning the blood cultures negative. However, the bacteraemia re-occurred after the discontinuation of phage therapy (Duplessis et al., 2018). It is however felt that commonly approved guidelines for application of phages is warranted in order to compare the efficacies of various phage treatments.

Several clinical trials have demonstrated the safety of phages and phage lytic enzymes which are in agreement with the studies carried out in animal models or as reported in numerous case studies. Although, many phase I and/or phase II clinical trials to demonstrate the efficacy of phages against ESKAPE infections have been registered in the past few years, the number of well-documented and completed trials are too low to draw meaningful conclusions (Sybesma et al., 2018). Moreover, the number of patients enrolled for the trials have severely limited the conclusions. A notable example of this is “PhagoBurn,” a multicentric randomized single blind and controlled clinical study on phage therapy to treat burn wound infections caused by *P. aeruginosa* and *E. coli* (ClinicalTrials.gov registry, NCT02116010). Twenty seven patients were enrolled for the trial which resulted in being too low from the pre-calculated 220 patients expected to give statistically significant results. Restrictive patient inclusion criteria, shorter duration of patient enrolment, and low incidence of burn wound infection during the period of study were described as possible reasons for the very few eligible patients (Servick, 2016). The 27 patients were randomly assigned to the phage treatment group and safety control group for further investigation. A cocktail of 12 phages (PP1131) with lytic activity against *P. aeruginosa* were added to an alginate template that was applied directly to the wound of the treated group. A diluted phage cocktail was used due to its high endotoxin content. The control group received a topical application of the standard care treatment (1% sulfadiazine silver). Few adverse events were reported both in phage treated and safety control group. The trial, however, was terminated prematurely due to insufficient efficacy of PP1131. A supporting study showed that the bacteria isolated from patients with the failed treatment were resistant to low phage doses used for this study. This, however, was the first Randomised Clinical Trial (RCT) performed following both good manufacturing practices as well as good clinical practices and was approved by three national health regulators (Belgium, France, and Switzerland). Further studies with increased phage concentration and higher number of participants are needed (Jault et al., 2019). In case of phage lytic enzymes, the first-in-human phase I clinical trial study for phage endolysin-based candidate drug SAL200, after a single intravenous administration among healthy volunteers showed no clinically significant detrimental effect (Youn Jun et al., 2017).

The other recent ongoing trial includes a randomized placebo controlled double blind clinical trial (ClinicalTrials.gov registry, NCT03140085) to study the efficacy and safety of commercially available Pyophages for treating UTI infections in patients planned for transurethral resection of prostate. Eighty-one patients were expected to enroll for this interventional study design planned between Nov 2015–Dec 2018 that may provide necessary insights into this potentially transforming alternative treatment option (Leitner et al., 2017). Similarly, another multicentric, randomized, two-parallel group, double blind controlled trial expected to enroll 60 patients was registered (ClinicalTrials.gov registry, NCT02664740) to compare efficacy of anti-staphylococcal phages verses standard treatment and placebo for diabetic foot ulcers infected by *S. aureus*. This study has not yet begun recruiting patients.

To further overcome limitations of phage therapy, phages can be combined with antibiotics which may show synergistic action by making either a phage or antibiotic or both to act more effectively (Table 3). Reduction in the formation of bacterial biofilms has also been reported when antibiotic treatment is used in combination with phages (Jo et al., 2016; Chaudhry et al., 2017). Endolysin produced by bacteriophages, proves to be more beneficial than lysing cell wall and facilitating entry of the antibiotic inside the bacteria (Rios et al., 2016). Phage PEV20 and ciprofloxacin exhibited a synergistic effect *in vitro* against *P. aeruginosa* (Lin Y. et al., 2018). Another interesting study reported that *P. aeruginosa*, while developing phage resistance when under attack by OMKO1 phage, changed the efflux pump mechanism which ultimately increased the sensitivity of *P. aeruginosa* to antibiotics. Such an approach creates a win-win situation causing the killing of the bacteria either through phage or by antibiotic action (Chan et al., 2016). Similarly, it has been reviewed that such changes in the surface receptors of any bacterium may also reduce its virulence (Rios et al., 2016). A case study reported OMKO1 phage in combination with ceftazidime could successfully treat a complicated postoperative *P. aeruginosa* infection in a patient who underwent an aortic arch replacement surgery (Chan et al., 2018). The success or failure of phage antibiotic combination therapy is still in a state of immaturity as the mechanisms involved in synergy have not been fully understood and the data of *in vivo* models and case reports is scarce. Further investigations are warranted in order to make any conclusive remarks.

## Antimicrobial Peptides (AMPs) in Therapy

Antimicrobial peptides (AMPs) are short, positively charged, diverse host defense oligopeptides produced by all living forms including protozoa, bacteria, archaea, fungi, plants, and animals (Wang et al., 2010). They show a broad spectrum of activity against a wide range of pathogens. The capacity of AMPs to interact with bacterial cell membrane and thereby cause cell lysis makes them a potential alternative to combat MDR pathogens (Berglund et al., 2015). Furthermore, in contrast to conventional antibiotics, AMPs physically damage the bacterial cell through electrostatic interactions thereby making it difficult for bacteria to develop resistance against AMPs (Pfalzgraff et al., 2018).

Considering the critical status of ESKAPE pathogens, several attempts have been made to find out AMP based effective therapeutics. To date, there are numerous natural as well as bioengineered AMPs reported to show *in vitro* (Björn et al., 2016; Cappiello et al., 2016; Liu et al., 2017; Gandt et al., 2018; Irani et al., 2018; Téllez et al., 2018) as well as *in vivo* (Björn et al., 2016; Liu et al., 2017) antimicrobial, antibiofilm, anti-inflammatory, and wound healing abilities with minimum cytotoxicity. Histatin 5, a natural histidine rich cationic human salivary peptide, is an example. This peptide shows a strong *in vitro* anti-biofilm as well as potent bactericidal activity (≥70%) against ESKAPE (Du et al., 2017). Similarly, a *de novo*-engineered cationic peptide, WLBU-2, and a natural AMP LL-37 at 1/3X MIC has demonstrated 90% biofilm inhibition as compared to that shown by antibiotics such as tobramycin, ciprofloxacin, ceftazidime, and vancomycin at 1X MIC (Lin Q. et al., 2018). In 2017, Gaglione et al. examined human ApoB derived recombinant peptides namely r(P)ApoB_L_ and r(P)ApoB_S._ Both peptides showed effective *in vitro* wound healing, anti-inflammatory, antimicrobial, and antibiofilm properties against MDR strains of *S. aureus* and *P. aeruginosa*.

Similar to their remarkable *in vitro* properties, AMPs also exhibit promising *in vivo* activity against ESKAPE. For example, peptide HLR1r, a structural derivative of human milk protein, lactoferrin, at very low concentration (5 mg/kg) was found to show anti-infectivity against MRSA infected wound excision model in rat along with *in vitro* anti-inflammatory and non-cytotoxic effects suggesting use of HLR1r in topical formulation to treat skin infections (Björn et al., 2016). PT-13 a peptide derived from seeds and leaves of *Populus trichocarpa* crude extract also demonstrated effective *in vivo* antibacterial activity in *S. aureus* infected *G. mellonella* model (Al Akeel et al., 2018). In another instance, a synthetic analog of Feleucin-K3 has shown to clear *P. aeruginosa* induced bacteremia in mice model with good stability and very low cytotoxicity (Xie et al., 2018). Also, a hydrogelformulation containing K-11, a hybrid peptide of melittin, cecropin A1 and magainin-2 has shown to possess wound healing ability against *A. baumannii* infected murine excision model proposing its possible use as a topical anti-infective therapeutic agent (Rishi et al., 2018).

Over the past decades, intense efforts taken by the scientific community and pharmaceutical industries together has made it possible to introduce certain peptides such as vancomycin, telavancin, telaprevir, teicoplanin, enfuvirtide, daptomycin, dalbavancin, bacitracin etc. for clinical use (Gomes et al., 2018). A clinical trial conducted on rabbits and humans using the peptide melamine proved it to be a stable and safe antibacterial coating for eye lenses (Dutta et al., 2014). Similarly, Pexiganan (analog of magainin), LL-37 (analog of human cathelicidin peptide), hLF1-11, and PXL-01 (derivatives of human milk protein), Novexatin (derivative of human defense peptide), Iseganan (derivative of porcine leukocytes), PAC-113 (derivative of human saliva histatin-3 peptide) etc. are few examples of AMPs which also are under clinical trials (Mahlapuu et al., 2016).

Unfortunately, such a low number of AMPs seeking clinical approval is quite discouraging. Despite their successful *in vitro* and/or *in vivo* broad-spectrum activities, numerous AMPs have not yet crossed the hurdle of clinical trial. Amongst the few challenges that hamper the *in vivo* efficacy of AMPs are their cytotoxicity to mammalian cells, liability to degradation by tissue proteases, loss of activity at low salt concentrations or in presence of plasma proteins and higher production cost (Mahlapuu et al., 2016; Rios et al., 2016). The issue of peptide degradation can be solved by structural modification of AMPs such as addition of non-natural amino acids or their D-isomers, peptide cyclisation, acetylation, and amidation of N-terminus. Introduction of peptide mimetics or the use of suitable delivery system like liposome encapsulation can be done to improve their stability and reduce toxicity (Seo et al., 2012; Reinhardt and Neundorf, 2016). Additionally, efficiency of AMPs can be enhanced by combining AMPs with antibiotics (Gaglione et al., 2017; Zheng et al., 2017; Pletzer et al., 2018) or nanoparticles (Chaudhari et al., 2016; Kuo et al., 2016). Otvos et al. (2018), reported a synergestic effect of A3-APO, a proline-rich AMP, and colistin when studied in a *K. pneumoniae* infected bacteremia mice model. The basis for *in vitro* decrease in MIC of A3-APO when combined with colistin can be explained by considering the fact that colistin kills bacteria by interfering with bacterial membrane assembly and, therefore, slight reductions in bacterial membrane integrity potentiate A3-APO antibacterial action. Surprisingly, the same combination also showed a 100% survival in mice. This observation can be a direct consequence of A3-APO ability to induce immune augmentation or the deactivation of bacterial toxins. Enhanced *in vitro* bactericidal activity against *S. aureus* was also found in the case where LL-37, a human cathelicidin peptide, combined with gold nanoparticles as compared to vancomycin alone (Wang et al., 2018). In this case, gold nanoparticles increased the local density of positive charges and peptide mass and thereby enhanced the bactericidal properties of LL-37.

To summarize, owing to their *in vitro* and *in vivo* broad-spectrum antibacterial activities AMPs offer a hopeful alternative to conventional therapeutics. However, to overcome challenges in developing a safe, stable and efficient commercial product, a thorough understanding of their structure and interaction with bacterial as well as host cells is still needed. It will also be helpful to find better AMP formulation strategies to obtain maximum therapeutic actions. Overall, considering the extensive research being carried out on different AMPs against various infectious agents, the future of peptide based commercial drug formulations looks hopeful.

## Photodynamic Light Therapy

Antimicrobial light therapy, either alone or combined with a photosensitizer (PS), results in a photooxidative stress response that leads to microbial death. Excitation of PS with light of an appropriate wavelength leads to formation of an excited triplet state. An excited PS can transfer electrons or energy to biomolecules or molecular oxygen, resulting in the formation of reactive oxygen species (ROS) or singlet oxygen radicals, which are toxic to cellular targets such as nucleic acids, proteins and lipids (Mai et al., 2017; Yang M.-Y. et al., 2018). Some of the most frequently used PSs include phenothiazinium derivatives (methylene blue, toluidine blue), xanthine derivatives (rose bengal), porphyrin, chlorin, or fullerene derivatives amongst many others (Abrahamse and Hamblin, 2016; Cieplik et al., 2018). Antimicrobial photodynamic therapy is widely used for treating dental, skin, and soft tissue infections. For a more detailed description of the current state and future prospects of light therapy with respect to the various photosensitisers, light sources, and methods used, mechanism of antimicrobial action or antibiofilm potential, the reader may be referred to the excellent reviews published recently (Cieplik et al., 2018; Hu et al., 2018; Tomb et al., 2018; Wozniak and Grinholc, 2018). However, none of these reviews have especially focused on *in vivo* studies of aPDT against ESKAPE pathogens.

There has been extensive research on designing the PSs so as to improve their pharmaceutical potential. An ideal PS used for antimicrobial therapy should have greater permeability to cross the microbial cell wall/cell membrane, selective toxicity toward the microbial cell with minimal or no damage to the host tissue and an absorption coefficient appropriate for effective penetration at the site of action. The PS chosen should not have a long half-life which causes prolonged photosensitization in the host cells even after the infection is cured. It should also not be effluxed out by the microbial efflux systems (Cieplik et al., 2018; Hu et al., 2018; Tomb et al., 2018; Wozniak and Grinholc, 2018). Efficacy of aPDT also depends on the light fluence, PS concentration and treatment time (Tomb et al., 2017; Sueoka et al., 2018; Ullah et al., 2018).

PSs chosen preferably have a large absorption coefficient in the visible spectrum, especially in the long wavelength (red near infrared) region, to allow effective penetration of light in the infected tissue (Table 2). Many researchers have attempted to improve the availability of PS by potentiating or functionalizing it with other molecules including galactose, amino acids, efflux pump inhibitors, potassium iodide, EDTA etc. A variety of PSs functionalized with addends are used to target ESKAPE pathogens. A boron-dipyrrolemethene (BODIPY)-based polygalactose, named pGEMA-I (7.3 kDa) with increased water solubility was used to demonstrate antibacterial and antibiofilm activity against *P. aeruginosa*, without much affecting the viability of normal cells. It was demonstrated that the selective recognition of the pathogen was due its carbohydrate binding lectin protein (LecA) which interacted with the galactose moiety of the PS (Zhao et al., 2018). C60-fullerene (LC16) bearing deca-quaternary chain and deca-tertiary-amino groups facilitates electron-transfer reactions via the photoexcited fullerene for antimicrobial effect studied in *A. baumannii* and *S. aureus* (Huang et al., 2014; Zhang et al., 2015). Another drawback of aPDT is that the ROS generation may cease after the light irradiation is turned off thus allowing un-killed bacteria to re-grow. Potentiating aPDT with potassium iodide allows the formation of iodine/tri-iodide that may remain active in the wound for a longer duration sufficient enough to prevent bacterial re-growth (Zhang et al., 2015; Wen et al., 2017).

*In vitro* studies have shown that blue light (aBL) has a broad spectrum antibacterial and antibiofilm activity against all six ESKAPE members (Halstead et al., 2016). *In vivo* data also corroborated this finding and further confirmed that using a low penetrating blue light of 415 ± 10 nm should be a preferred choice of treatment in case of topical wound infections as it causes minimal damage to the uninfected tissue cells below (Amin et al., 2016; Wang et al., 2017; Katayama et al., 2018). Some studies additionally report that an exogenous PS may not be required (Amin et al., 2016; Wang et al., 2017). Their finding was supported by experimental data showing that the endogenous porphyrins present in the bacterial cell membrane play a role in triggering the photoxidative response (Amin et al., 2016). aBL using 5-aminolevulinic acid with disodium EDTA (ALA-EDTA/2Na) had antibacterial and antibiofilm potential thus showing significant wound healing of *P. aeruginosa* infected cutaneous ulcers in mice model (Katayama et al., 2018). However, the role of EDTA in increasing the antibacterial action of aBL needs further investigation. The physiological mechanism behind wound healing was studied in a *S. aureus* infected burn model in mice revealing that the enhanced levels of factors promoting angiogenesis and epithelial regeneration (bFGF, TGFβ1, and VEGF) led to the inhibition of inflammatory factors (TNFα and IL6) in the aPDT treated group as compared to the control (Mai et al., 2017).

Sueoka et al. (2018) studied the time dependant effect of aPDT-TON 504 on *P. aeruginosa* and showed that a repeated exposure to light emitting diode (LED) enhanced the inhibitory effect on bacterial growth. This enhanced effect was possibly because the bacteria that survived the initial aPDT were injured by singlet oxygen generated due to excitation of remaining photosensitizer. On the contrary there are also a few reports investigating the development of resistance/tolerance to aBL. It was observed that initial exposure to low doses of aBL increased the tolerance of methicillin susceptible *S. aureus* (MSSA) to subsequent doses of high intensity aBL-405 nm. It is likely that increased tolerance to high intensity light may be due to up-regulation of bacterial stress responses which needs detailed investigations. However, a second set of experiment showed that repeated sub-lethal exposures of 405 nm light indicate no evidence of tolerance in *S. aureus* (Tomb et al., 2017).

A considerable amount of literature has been published on combined efficacy of aPDT and antibiotics demonstrated *in vitro* (El-Azizi and Khardori, 2016; Ronqui et al., 2016). Synergistic effects of aPDT-antibiotic combination resulted in inactivation of several virulence factors in *P. aeruginosa* isolates (Fila et al., 2017). aPDT when used in combination with vancomycin prolonged the survival of *Galleria mellonella* infected with a vancomycin resistant strain of *E. faecium* as compared to either of the two therapies used alone (Chibebe Junior et al., 2013). Wozniak and Grinholc (2018) in their comprehensive review analysis have, however, pointed out that most of the aPDT carried out in combination with antibiotics lack a standard methodology followed to evaluate the synergistic effect.

One of the strategies for improved PS delivery involves the use of nanoparticles which are co-administered to allow the PS entry across the membrane or for a synergistic ROS response resulting in an antimicrobial action. A combination of tetrasulfonated hydroxyl aluminum phthalocyanine (AlPcS_4_) and bimetallic gold/silver nanoparticles (Au/Ag-NPs) synthesized using a cell-free filtrate of *Aureobasidium pullulans* showed significantly higher killing as compared to the agents used individually (Maliszewska et al., 2018). Au/Ag-NPs possibly disrupted the cell membrane allowing enhanced uptake of the PS, AlPcS_4_. *In vitro* and *in vivo* studies demonstrated that AgNPs used in combination with blue light showed synergistic antimicrobial and antibiofilm activities against *P. aeruginosa* infection. Interestingly, this combination was also effective for the treatment of a chronic wound caused by mixed infection in a horse (Nour El Din et al., 2016). More recently, an *in vitro* study carried out using *A. baumannii* isolates, demonstrated that the antibacterial action of ZnO-NPs with blue light irradiation was due to their ability to damage the cytomembrane but not DNA (Yang M.-Y. et al., 2018).

Over expressing multidrug efflux pumps, commonly found in resistant pathogens, are reported to affect the intracellular concentration of PS used in aPDT, thus limiting its action (Tegos and Hamblin, 2006). Tegos et al. (2008) carried out *in vitro* experiments by co-incubating various combinations of PS with efflux pump inhibitors to select the best combination showing antibacterial activity. Photodynamic killing mediated by toluidine blue (TBO), when used in combination with PaβN, or INF271 (as EPIs) was most effective against *P. aeruginosa* and *S. aureus* isolates, respectively. Similarly, an *in vitro* study demonstrated that verapamil, an efflux pump inhibitor, when combined with aPDT, required a lower light dose for effective antibacterial, and antibiofilm action against *S. aureus* (de Aguiar Coletti et al., 2017). In further development of such an approach, it was recently demonstrated that INF55-(Ac)en–MB, synthetic antimicrobial hybrids designed by covalently linking a PS (methylene blue) to efflux pump inhibitors (INF55 and INF271) were more effective in treating wound infections caused by *S. aureus* or *A. baumannii* studied in mice models (Rineh et al., 2017, 2018).

It is also worth noting that synergistic effect of aPDT when used in combination with antimicrobial peptide was also demonstrated recently (de Freitas et al., 2018; Nakonieczna et al., 2018). Aurein 1.2 augmented the aPDT activity mediated by methylene blue or chlorin-e6 against strains of *S. aureus, A. baumannii* and more importantly against vancomycin resistant *E. faecium*, whereas the AMP aPDT combination with curcumin (as PS) had no effect thus revealing a PS-dependent mechanism (de Freitas et al., 2018). Later, Nakonieczna et al. (2018) carried out a similar study showing synergistic effect of rose bengal-aPDT with two synthetic AMPs, CAMEL, and pexiganan against 35 isolates of *P. aeruginosa*. Notably, it was also shown that this combination was non-toxic to human keratinocytes. Conjugates such as ZnPc(Lys)_5_ (a zinc phthalocyanine derivative coupled with pentalysine) also showed a synergistic antibacterial action which was sufficient to heal *S. aureus* wound infection in mice models (Ullah et al., 2018).

Overall, photodynamic therapy appears to be a promising option for treatment of infections caused due to ESKAPE pathogens, particularly effective in topical applications. aPDT co administered or conjugated with antibiotics, antimicrobial peptides, nanoparticles, or efflux pump inhibitors show a synergistic effect. However, it is difficult to compare efficacy between different combinatorial approaches due to lack of uniform methodologies. More studies on investigation of toxicity and biocompatibility of various combinations should be investigated using *in vivo* models for translating them into clinical practice.

## Silver Nanoparticles in Therapy

Nanomedicine is one of the emerging branches for treating drug resistant pathogens. Metal nanoparticles have wide biomedical applications as antimicrobial agents due to their unique physical and chemical properties (Beyth et al., 2015; Hemeg, 2017). Amongst metal nanoparticles, silver nanoparticles (AgNPs) synthesized using physical, chemical or biological methods have shown promising antibacterial activity due to their multi-targeted approach which reduces the probability of resistance (Möhler et al., 2018; Siddiqi et al., 2018). AgNPs act by releasing Ag^+^ ions which results in disruption of electron transport or signal transduction pathway or leads to generation of ROS, ultimately damaging important biomolecules such as cell wall, cell membrane, cellular DNA, and/or proteins (Dakal et al., 2016; Qayyum et al., 2017).

AgNPs act by inhibiting or disrupting planktonic cells as well as biofilms of MDR pathogens. Even though earlier reports have suggested the cytotoxic effects of AgNPs (Mohanty et al., 2012), recently *in vitro* and *in vivo* studies have demonstrated the safe usage of AgNPs (Möhler et al., 2018). AgNPs synthesized using aqueous leaf extract of *Corchorus capsularis* exhibited antibacterial activity against *S. aureus* and *P. aeruginosa* and were found to be non-toxic to mouse fibroblast cells (Kasithevar et al., 2017). Electrochemically synthesized AgNPs showing antimicrobial activity against planktonic and biofilm forming *P. aeruginosa* strain were non-toxic to *G. mellonella* larvae model (Pompilio et al., 2018). Recently, sunlight mediated AgNPs synthesized using *Capsicum annuum* was tested in *S. aureus* infected zebra fish model which proved to be effective in inhibiting biofilm formation. Histological studies revealed that they are non-toxic and hence can be tested for efficacy in higher mammalian *in vivo* models (Lotha et al., 2018). A single-blind clinical trial (Clinical Trial Registration: NCT01243320 and NCT01405794) carried out in 60 healthy human volunteers, showed that commercial AgNPs when administered orally at dose of 10 and 32 ppm and monitored over 14 days were found to be non-toxic. The study revealed no significant changes in metabolic, hematologic and pro-inflammatory responses as well as no morphological changes in vital organs (Munger et al., 2014).

One of the widely explored applications of AgNPs is their use in the form of composite dressings or hydrogels for treatment of topical wound infections. AgNPs incorporated in chitosan composite dressings offer sustained release of Ag^+^ ions at low dosage which are non-toxic to fibroblast cells. Studies in mice models suggested that AgNPs/chitosan composite dressings and low molecular weight chitosan-coated silver nanoparticles were effective in reducing bacterial load, were non-toxic and biocompatible, had low absorption in body and promoted better wound healing against *S. aureus* and *P. aeruginosa* (Liang et al., 2016; Peng et al., 2017). Similarly, studies using three other polymer dressings made of chitosan, nylon, and collagen incorporated with AgNPs exhibited *in vitro* antibacterial activities against ESKAPE pathogens (Radulescu et al., 2016; Rath et al., 2016; Ding et al., 2017). These dressings did not exhibit inflammatory responses, showed re-epithelization of cells and better wound contraction leading to accelerated wound healing in mice models. Sodium carboxymethyl cellulose hydrogel loaded with polyethylene glycol coated AgNPs showed antibacterial activity, re-epithelization, and wound healing in MRSA infected mice model (Mekkawy et al., 2017). Similarly, topical application of nanosilvernanohydrogels in combination with *Aloe vera* accelerated wound contraction and enhanced wound healing due to the moist environment provided by *Aloe vera* (Anjum et al., 2016). In yet another formulation, AgNPs coated on to MCM-41 type mesoporous silica nanoparticles prevented their aggregation and allowed sustained release of Ag^+^ ions displaying a long-term antibacterial activity against *S. aureus*. These antibacterial nanofibrous membranes could reduce inflammatory response and accelerate wound healing in wistar rats (Dong et al., 2016). A randomized clinical trial was carried out to test the antibacterial effect of two silver dressings and their healing time in burn patients. It was demonstrated that the hydrofiber silver dressing (Aquacel^R^) was preferred over the nanocrystalline silver dressing (Acticoat^TM^) due to reduction of bioburden, quick wound healing, ease of using, comfort to the patients, and low cost (Verbelen et al., 2014). On the other hand Acticoat^TM^ showed complete wound healing within 12 weeks in 64% of the patients with leg ulcers as compared to those who were treated with Iodosorb dressings (cadexomer iodine) (Miller et al., 2010).

Polymer-based nanomaterials and metal NPs are used in antimicrobial coatings on surface of medical devices, such as catheters and implants for prevention of infections. AgNPs when embedded in electrospun hyaluronic acid/polycaprolactonenanofibrous membranes coated on flexor tendon animal models prevented bacterial infection during the early postsurgical period (Chen et al., 2015; Shalumon et al., 2018). Likewise, implants coated with nanocomposite layer of polysaccharide 1-deoxylactit-1-yl chitosan and AgNPs in a mini-pig animal model showed good biocompatibility with the bone tissue (Marsich et al., 2013). All these studies demonstrated that the entrapped AgNPs allowed controlled release of Ag^+^ ions displaying prolonged antibacterial and antibiofilm action as well as reduced inflammatory responses. A randomized clinical trial demonstrated the efficacy of triple-lumen central venous catheters impregnated with AgNPs (AgTive) which showed reduced bacterial colonization as compared to conventional catheters in intensive care unit patients (Antonelli et al., 2012).

Repeated exposure of AgNPs at sub inhibitory concentrations may lead to resistance in bacterial pathogens. To overcome this limitation, a combination of AgNPs with antibiotics has been suggested in order to increase the therapeutic efficacy of either, resulting in reduction of dose and hence toxicity. *In vitro* studies demonstrating AgNPs co-incubated with different antibiotics showed synergistic antibacterial activities against ESKAPE (Ghosh et al., 2012; Panáček et al., 2015; Golińska et al., 2016; Habash et al., 2017; Singh et al., 2018; Wypij et al., 2018). Synergistic antibacterial activity of AgNPs in combination with polymyxin B was demonstrated in *A. baumannii* infected mouse model with 60% survival rate as compared to the controls treated with antibiotic or AgNPs alone (Wan et al., 2016).

Despite the use of AgNPs as a potential therapeutic agent, literature survey indicates a paucity of data obtained from *in vivo* studies carried out to test the toxicity, efficacy, pharmacokinetic, and immuno-modulatory response of the AgNPs. Further investigations through well-defined studies and clinical trials will lead to applications of AgNPs in wound dressings or medical devices.

## Concluding Remarks

There is an urgent need to restock our armamentarium of antimicrobials in order to stay ahead of the ever rising drug resistant ESKAPE pathogens. There is an insufficiency of effective antibiotic combinations in addition to the dry pipeline of new drugs. Huge efforts have been taken to use antibiotics in combination with adjuvants targeting important metabolic mechanisms/pathways contributing to drug resistance (permeablisers, lactamase inhibitors, efflux pump inhibitors, quorum sensing inhibitors, toxin inhibitors etc.) The modest success received to date with such antibiotic-adjuvant combinations has paved way to explore other alternative strategies to combat drug resistance. There is a significant rise in the interest shown by the scientific community to use novel therapeutic agents such as phages, antimicrobial peptides, metal nanoparticles, and photodynamic light which, although, have some limitations as discussed above. Some of the commonly described limitations of these therapies include stability and toxicity of the therapeutic agent, its targeted delivery at the site of infection, or immune response developed by the host against the therapeutic agent. Ongoing research has therefore led to further develop or modify these novel therapeutic agents or therapies so as to surmount the limitations as well as to overcome the barriers of bacterial resistance.

This review summarizes studies that demonstrate potential alternative therapies using *in vivo* models some of which have extended further to the level of clinical trials. The interest in phage therapy to treat bacterial infections is fast growing leading to development of commercial preparations such as “Stafal,” “Sextaphage,” “PhagoBioDerm,” and “Pyophage” against MDR pathogens. Similarly, use of silver nanoparticles as antibiofilm coatings in surgical implants, antimicrobial agents in topical applications or as formulations in wound dressings has shown promising activities in animal models. Clinical trials using commercially available nanosilver coated dressings (Acticoat^TM^, Aquacel^R^) or catheters (AgTive) is another noteworthy advancement. AMPs have received great attention due to their broad spectrum activity; however, they have shown limited pharmaceutical potential due to their toxicity, stability, and production costs. Photodynamic light therapy which is widely used for cancer therapy has also been demonstrated to be an effective strategy for clearing wound infections. However, additional studies demonstrating the efficacy and safety of these therapeutic agents against ESKAPE infections are desired. Similarly, randomized clinical trials would enable these therapeutic agents to cross the regulatory hurdles and find application in clinical practice.

It was observed that, majority of these studies have used animal models infected by *S. aureus, A. baumannii*, and *P. aeruginosa* to test the efficacy of the therapeutic agent. The probable reason for this could be that these pathogens mostly cause topical infections (wound, burn and abscess) and because majority of the limitations (targeted delivery, stability, immune response, toxicity etc.) described for each therapy can be minimized though not avoided in such models. It would be important to study the effect of these therapeutic agents against systemic infections caused by ESKAPE members. It was also observed that, the methods used for estimating efficacy of any therapeutic agent were not uniform. Table 2 reveals that, the *in vivo* efficacy of various therapies is given either in terms of log or percent reduction of microbial load or as percent survival of the infected host (animal model). The methods followed to estimate the reduced pathogen loads as well as dosages used for treatment also vary. It is therefore not appropriate to compare these studies to identify the best therapeutic agent/therapy against any ESKAPE member.

In addition to the growing concern in searching and evaluating the clinical potential of the above discussed alternative therapies, research on combinatorial approach, based on the synergistic action of two or more therapies is also gaining attention. Most commonly studied combinations involve use of a therapeutic agent/ therapy (phage, aPDT, AMP, or AgNP) in combination with antibiotic/s or in some cases with an efflux pump inhibitor or quorum sensing inhibitor. Another interesting option used was the combination of the therapeutic agents in a conjugate or hybrid (antibiotic-antibiotic, antibiotic-EPI, PS-AMP, PS-EPI etc.) for an increased efficacy against the pathogen. Most of these studies demonstrated that the combinatorial approach helped overcome the limitation caused by individual therapeutic agent. For example, an antibiotic combined with an efflux pump inhibitor or a photosensitizer conjugated with an AMP improved their entry and retention into the target pathogen for enhanced antimicrobial action. Similarly, combinations of antibiotics with nanoparticles or AMPS reduced the toxicity caused by these agents which were required at high dosages when used alone. The synergistic action allows for an increased bioavailability of the drug or therapeutic agent, broad antimicrobial spectrum, reduced toxicity, and decreased chances of development of resistance. However, Table 3 shows that most of the studies demonstrating potential of combinatorial approach are currently based on *in vitro* evaluation only. Data supporting the potential of a combinatorial therapy with respect to the mechanism of synergy, its *in vivo* efficacy, toxicity, and immune response is scarce and needs further investigation.

Finally, cost effectiveness of the above described therapeutic agents/therapy over the conventional antimicrobial agents also play a crucial role in their clinical application. Production cost for any therapeutic agent will strongly depend on the various regulatory hurdles they pass to come into clinical practice. An agent or therapy which is too expensive may not be a preferred choice for treatment in under developed countries or weaker economies thus leading to over-use of conventional antimicrobials thus contributing to the growing drug resistance.

To conclude, a uniform research methodology used to test the efficacy of these therapeutic agents in accordance with well-defined standards will make it possible to reliably compare the data presented by various research groups. Well-performed clinical trials of these therapeutic agents used as monotherapy or as a combinatorial approach will allow us to derive the real potential of these therapeutic combinations for being translated into clinical practice.

## Author Contributions

KP conceived the concept and edited. MM, EK, SK, and MT wrote and edited the manuscript and agreed for submission.

### Conflict of Interest Statement

The authors declare that the research was conducted in the absence of any commercial or financial relationships that could be construed as a potential conflict of interest. The reviewer RP declared a shared affiliation, with no collaboration, with the authors to the handling editor at the time of the review.

## References

[B1] Abedon S. T. Kuhl S. J. Blasdel B. G. Kutter E. M. (2011). Phage treatment of human infections. Bacteriophage 1, 66–85. 10.4161/bact.1.2.15845 22334863 PMC3278644

[B2] Abrahamse H. Hamblin M. R. (2016). New photosensitizers for photodynamic therapy. Biochem. J. 473, 347–364. 10.1042/BJ20150942 26862179 PMC4811612

[B3] Ahmed A. Azim A. Gurjar M. Baronia A. K. (2014). Current concepts in combination antibiotic therapy for critically ill patients. Indian J. Crit. Care Med. 18, 310–314. 10.4103/0972-5229.132495 24914260 PMC4047693

[B4] Akram F. E. El-Tayeb T. Abou-Aisha K. El-Azizi M. (2016). A combination of silver nanoparticles and visible blue light enhances the antibacterial efficacy of ineffective antibiotics against methicillin-resistant *Staphylococcus aureus* (MRSA). Ann. Clin. Microbiol. Antimicrob. 15, 48. 10.1186/s12941-016-0164-y 27530257 PMC4988001

[B5] Al Akeel R. Mateen A. Syed R. Alqahtani M. S. Alqahtani A. S. (2018). Alanine rich peptide from *Populus trichocarpa* inhibit growth of *Staphylococcus aureus* via targetting its extracellular domain of Sensor Histidine Kinase YycGex protein. Microb. Pathog. 121, 115–122. 10.1016/j.micpath.2018.05.010 29758266

[B6] Alipour N. Karagoz A. Taner A. Gaeini N. Alipour N. Zeytin H.. (2017). Outbreak of hospital infection from biofilm-embedded pan drug-resistant *Pseudomonas aeroginosa*, due to a contaminated bronchoscope. J. Prev. Med. 2:1. 10.21767/2572-5483.100014 29225413

[B7] Allegranzi B. Nejad S. B. Combescure C. Graafmans W. Attar H. Donaldson L.. (2011). Burden of endemic health-care-associated infection in developing countries: systematic review and meta-analysis. Lancet 377, 228–241. 10.1016/S0140-6736(10)61458-4 21146207

[B8] Amin R. M. Bhayana B. Hamblin M. R. Dai T. (2016). Antimicrobial blue light inactivation of *Pseudomonas aeruginosa* by photo-excitation of endogenous porphyrins: *in vitro* and *in vivo* studies. Lasers Surg. Med. 48, 562–568. 10.1002/lsm.22474 26891084 PMC4914480

[B9] Anjum S. Gupta A. Sharma D. Gautam D. Bhan S. Sharma A.. (2016). Development of novel wound care systems based on nanosilver nanohydrogels of polymethacrylic acid with *Aloe vera* and curcumin. Mater. Sci. Eng. C. Mater. Biol. Appl. 64, 157–166. 10.1016/j.msec.2016.03.069 27127040

[B10] Antonelli M. De Pascale G. Ranieri V. M. Pelaia P. Tufano R. Piazza O.. (2012). Comparison of triple-lumen central venous catheters impregnated with silver nanoparticles (AgTive®) vs. conventional catheters in intensive care unit patients. J. Hosp. Infect. 82, 101–107. 10.1016/j.jhin.2012.07.010 22938728

[B11] Aoki N. Ishii Y. Tateda K. Saga T. Kimura S. Kikuchi Y.. (2010). Efficacy of calcium-EDTA as an inhibitor for metallo-β-lactamase in a mouse model of *Pseudomonas aeruginosa* pneumonia. Antimicrob. Agents Chemother. 54, 4582–4588. 10.1128/AAC.00511-10 20713659 PMC2976153

[B12] Babouee Flury B. Ellington M. J. Hopkins K. L. Turton J. F. Doumith M. Loy R.. (2016). Association of novel nonsynonymous single nucleotide polymorphisms in ampD with cephalosporin resistance and phylogenetic variations in ampC, ampR, ompF, and ompC in *Enterobacter cloacae* isolates that are highly resistant to carbapenems. Antimicrob. Agents Chemother. 60, 2383–2390. 10.1128/AAC.02835-15 26856839 PMC4808197

[B13] Balamurugan P. Hema M. Kaur G. Sridharan V. Prabu P. C. Sumana M. N.. (2015). Development of a biofilm inhibitor molecule against multidrug resistant *Staphylococcus aureus* associated with gestational urinary tract infections. Front. Microbiol. 6:832. 10.3389/fmicb.2015.00832 26322037 PMC4531255

[B14] Berglund N. A. Piggot T. J. Jefferies D. Sessions R. B. Bond P. J. Khalid S. (2015). Interaction of the antimicrobial peptide polymyxin B1 with both membranes of *E. coli*: a molecular dynamics study. PLoS Comput. Biol. 11:e1004180. 10.1371/journal.pcbi.1004180 25885324 PMC4401565

[B15] Bernal P. Molina-Santiago C. Daddaoua A. Llamas M. A. (2013). Highlight Antibiotic adjuvants: identification and clinical use. Microb. Biotechnol. 6, 445–449. 10.1111/1751-7915.12044 23445397 PMC3918149

[B16] Beyth N. Houri-Haddad Y. Domb A. Khan W. Hazan R. (2015). Alternative antimicrobial approach: nano-antimicrobial materials. Evid. Based Complement. Altern. Med. 2015:246012. 10.1155/2015/246012 25861355 PMC4378595

[B17] Biswas B. Adhya S. Washart P. Paul B. Trostel A. N. Powell B.. (2002). Bacteriophage therapy rescues mice bacteremic from a clinical isolate of vancomycin-resistant *Enterococcus faecium*. Infect. Immun. 70, 204–210. 10.1128/IAI.70.1.204-210.2002 11748184 PMC127648

[B18] Björn C. Mahlapuu M. Mattsby-Baltzer I. Håkansson J. (2016). Anti-infective efficacy of the lactoferrin-derived antimicrobial peptide HLR1r. Peptides 81, 21–28. 10.1016/j.peptides.2016.04.005 27155369

[B19] Bueno J. (2016). Antimicrobial adjuvants drug discovery, the challenge of avoid the resistance and recover the susceptibility of multidrug-resistant strains. J. Microb. Biochem. Technol. 8, 169–176. 10.4172/1948-5948.1000281

[B20] Cai Y. Yang D. Wang J. Wang R. (2018). Activity of colistin alone or in combination with rifampicin or meropenem in a carbapenem-resistant bioluminescent *Pseudomonas aeruginosa* intraperitoneal murine infection model. J. Antimicrob. Chemother. 73, 456–461. 10.1093/jac/dkx399 29149302

[B21] Caio C. Maugeri G. Zingali T. Gona F. Stefani S. Mezzatesta M. L. (2018). Extensively drug-resistant ArmA-producing *Acinetobacter baumannii* in an Italian intensive care unit. N. Microbiol. 41, 159–161. 29313866

[B22] Cappiello F. Di Grazia A. Segev-Zarko L.-A. Scali S. Ferrera L. Galietta L.. (2016). Esculentin-1a-derived peptides promote clearance of *Pseudomonas aeruginosa* internalized in bronchial cells of cystic fibrosis patients and lung cell migration: biochemical properties and a plausible mode of action. Antimicrob. Agents Chemother. 60, 7252–7262. 10.1128/AAC.00904-16 27671059 PMC5119005

[B23] Casey E. van Sinderen D. Mahony J. (2018). *In vitro* characteristics of phages to guide ‘real life' phage therapy suitability. Viruses 10:E163. 10.3390/v10040163 29601536 PMC5923457

[B24] Chadha P. Katare O. P. Chhibber S. (2017). Liposome loaded phage cocktail: enhanced therapeutic potential in resolving *Klebsiella pneumoniae* mediated burn wound infections. Burns 43, 1532–1543. 10.1016/j.burns.2017.03.029 28502784

[B25] Chan B. K. Abedon S. T. Loc-Carrillo C. (2013). Phage cocktails and the future of phage therapy. Future Microbiol. 8, 769–783. 10.2217/fmb.13.47 23701332

[B26] Chan B. K. Sistrom M. Wertz J. E. Kortright K. E. Narayan D. Turner P. E. (2016). Phage selection restores antibiotic sensitivity in MDR Pseudomonas aeruginosa. Sci. Rep. 6:26717. 10.1038/srep26717 27225966 PMC4880932

[B27] Chan B. K. Turner P. E. Kim S. Mojibian H. R. Elefteriades J. A. Narayan D. (2018). Phage treatment of an aortic graft infected with *Pseudomonas aeruginosa*. Evol. Med. Public Heal. 2018, 60–66. 10.1093/emph/eoy005 29588855 PMC5842392

[B28] Chan L. C. Basuino L. Diep B. Hamilton S. Chatterjee S. S. Chambers H. F. (2015). Ceftobiprole- and ceftaroline-resistant methicillin-resistant *Staphylococcus aureus*. Antimicrob. Agents Chemother. 59, 2960–2963. 10.1128/AAC.05004-14 25753637 PMC4394828

[B29] Chang R. Y. K. Chen K. Wang J. Wallin M. Britton W. Morales S.. (2018). Proof-of-principle study in a murine lung infection model of antipseudomonal activity of phage PEV20 in a dry-powder formulation. Antimicrob. Agents Chemother. 62:e01714-17. 10.1128/AAC.01714-17 29158280 PMC5786808

[B30] Chatterjee M. Anju C. P. Biswas L. Anil Kumar V. Gopi Mohan C. Biswas R. (2016). Antibiotic resistance in *Pseudomonas aeruginosa* and alternative therapeutic options. Int. J. Med. Microbiol. 306, 48–58. 10.1016/j.ijmm.2015.11.004 26687205

[B31] Chaudhari A. A. Ashmore D. Nath S. deb Kate K. Dennis V. Singh S. R.. (2016). A novel covalent approach to bio-conjugate silver coated single walled carbon nanotubes with antimicrobial peptide. J. Nanobiotechnol. 14:58. 10.1186/s12951-016-0211-z 27412259 PMC4944237

[B32] Chaudhry W. N. Concepcion-Acevedo J. Park T. Andleeb S. Bull J. J. Levin B. R. (2017). Synergy and order effects of antibiotics and phages in killing *Pseudomonas aeruginosa* biofilms. PLoS ONE 12:e0168615. 10.1371/journal.pone.0168615 28076361 PMC5226664

[B33] Chen C. Mangoni M. L. Di Y. P. (2017). *In vivo* therapeutic efficacy of frog skin-derived peptides against *Pseudomonas aeruginosa*-induced pulmonary infection. Sci. Rep. 7:8548. 10.1038/s41598-017-08361-8 28819175 PMC5561116

[B34] Chen C. H. Chen S. H. Shalumon K. T. Chen J. P. (2015). Dual functional core–sheath electrospun hyaluronic acid/polycaprolactone nanofibrous membranes embedded with silver nanoparticles for prevention of peritendinous adhesion. Acta Biomater. 26, 225–235. 10.1016/j.actbio.2015.07.041 26234491

[B35] Chen H. L. Su P. Y. Kuo S. C. Lauderdale T. L. Y. Shih C. (2018). Adding a C-terminal cysteine (CTC) can enhance the bactericidal activity of three different antimicrobial peptides. Front. Microbiol. 9:1440. 10.3389/fmicb.2018.01440 30002652 PMC6031733

[B36] Chen J. Novick R. P. (2009). Phage-mediated intergeneric transfer of toxin genes. Science 323, 139–141. 10.1126/science.1164783 19119236

[B37] Chhibber S. Kaur J. Kaur S. (2018). Liposome entrapment of bacteriophages improves wound healing in a diabetic mouse MRSA infection. Front. Microbiol. 9:561. 10.3389/fmicb.2018.00561 29651276 PMC5884882

[B38] Chibebe Junior J. Fuchs B. B. Sabino C. P. Junqueira J. C. Jorge A. O. C. Ribeiro M. S.. (2013). Photodynamic and antibiotic therapy impair the pathogenesis of *Enterococcus faecium* in a whole animal insect model. PLoS ONE 8:e55926. 10.1371/journal.pone.0055926 23457486 PMC3573038

[B39] Chuang L. Ratnayake L. (2018). Overcoming challenges of treating extensively drug-resistant *Acinetobacter baumannii* bacteraemic urinary tract infection. Int. J. Antimicrob. Agents. 52, 521–522. 10.1016/j.ijantimicag.2018.07.016 30081140

[B40] Cieplik F. Deng D. Crielaard W. Buchalla W. Hellwig E. Al-Ahmad A.. (2018). Antimicrobial photodynamic therapy – what we know and what we don't. Crit. Rev. Microbiol. 44, 571–589. 10.1080/1040841X.2018.1467876 29749263

[B41] Ciociola T. Giovati L. Giovannelli A. Conti S. Castagnola M. Vitali A. (2018). The activity of a mammalian proline-rich peptide against Gram-negative bacteria, including drug-resistant strains, relies on a nonmembranolytic mode of action. Infect. Drug Resist. 11, 969–979. 10.2147/IDR.S165179 30046246 PMC6054295

[B42] Cooper C. J. Koonjan S. Nilsson A. S. (2018). Enhancing whole phage therapy and their derived antimicrobial enzymes through complex formulation. Pharmaceuticals 11:E34. 10.3390/ph11020034 29671806 PMC6027540

[B43] Corbett D. Wise A. Langley T. Skinner K. Trimby E. Birchall S.. (2017). Potentiation of antibiotic activity by a novel cationic peptide: potency and spectrum of activity of SPR741. Antimicrob. Agents Chemother. 61, e00200–e00217. 10.1128/AAC.00200-17 28533232 PMC5527571

[B44] Coronado-Álvarez N. M. Parra D. Parra-Ruiz J. (2018). Clinical efficacy of fosfomycin combinations against a variety of gram-positive cocci. Enferm. Infecc. Microbiol. Clin. 37, 4–10. 10.1016/j.eimc.2018.05.009 29907368

[B45] Costley D. Nesbitt H. Ternan N. Dooley J. Huang Y. Y. Hamblin M. R.. (2017). Sonodynamic inactivation of gram-positive and gram-negative bacteria using a rose bengal-antimicrobial peptide conjugate. Int. J. Antimicrob. Agents 49, 31–36. 10.1016/j.ijantimicag.2016.09.034 27908581 PMC5191983

[B46] Dakal T. C. Kumar A. Majumdar R. S. Yadav V. (2016). Mechanistic basis of antimicrobial actions of silver nanoparticles. Front. Microbiol. 7:1831. 10.3389/fmicb.2016.01831 27899918 PMC5110546

[B47] Danis-Wlodarczyk K. Vandenheuvel D. Jang H. B. Briers Y. Olszak T. Arabski M.. (2016). A proposed integrated approach for the preclinical evaluation of phage therapy in Pseudomonas infections. Sci. Rep. 6:28115. 10.1038/srep28115 27301427 PMC4908380

[B48] de Aguiar Coletti T. M. S. F. de Freitas L. M. Almeida A. M. F. Fontana C. R. (2017). Optimization of antimicrobial photodynamic therapy in biofilms by inhibiting efflux pump. Photomed. Laser Surg. 35, 378–385. 10.1089/pho.2016.4246 28621579

[B49] de Breij A. Riool M. Cordfunke R. A. Malanovic N. de Boer L. Koning R. I.. (2018). The antimicrobial peptide SAAP-148 combats drug-resistant bacteria and biofilms. Sci. Transl. Med. 10:eaan4044. 10.1126/scitranslmed.aan4044 29321257

[B50] de Freitas L. M. Lorenzón E. N. Santos-Filho N. A. Zago L. H. P. Uliana M. P. de Oliveira K. T.. (2018). Antimicrobial photodynamic therapy enhanced by the peptide aurein 1.2. Sci. Rep. 8:4212. 10.1038/s41598-018-22687-x 29523862 PMC5844988

[B51] Defraine V. Verstraete L. Van Bambeke F. Anantharajah A. Townsend E. M. Ramage G.. (2017). Antibacterial activity of 1-[(2,4-Dichlorophenethyl)amino]-3-Phenoxypropan-2-ol against antibiotic-resistant strains of diverse bacterial pathogens, biofilms and in pre-clinical infection models. Front. Microbiol. 8:2585. 10.3389/fmicb.2017.02585 29312259 PMC5744096

[B52] Ding L. Shan X. Zhao X. Zha H. Chen X. Wang J.. (2017). Spongy bilayer dressing composed of chitosan-Ag nanoparticles and chitosan-*Bletilla striata* polysaccharide for wound healing applications. Carbohydr. Polym. 157, 1538–1547. 10.1016/j.carbpol.2016.11.040 27987866

[B53] Domalaon R. Idowu T. Zhanel G. G. Schweizer F. (2018). Antibiotic hybrids: the next generation of agents and adjuvants against gram-negative pathogens? Clin. Microbiol. Rev. 31, e00077–e00017. 10.1128/CMR.00077-17 29540434 PMC5967690

[B54] Domingo-Calap P. Delgado-Martínez J. (2018). Bacteriophages: protagonists of a post-antibiotic era. Antibiotics 7:66. 10.3390/antibiotics7030066 30060506 PMC6163168

[B55] Dong R. H. Jia Y. X. Qin C. C. Zhan L. Yan X. Cui L.. (2016). *In situ* deposition of a personalized nanofibrous dressing via a handy electrospinning device for skin wound care. Nanoscale 8, 3482–3488. 10.1039/C5NR08367B 26796508

[B56] Du H. Puri S. McCall A. Norris H. L. Russo T. Edgerton M. (2017). Human salivary protein histatin 5 has potent bactericidal activity against ESKAPE pathogens. Front. Cell. Infect. Microbiol. 7:41. 10.3389/fcimb.2017.00041 28261570 PMC5309243

[B57] Duplessis C. Biswas B. Hanisch B. Perkins M. Henry M. Quinones J.. (2018). Refractory *Pseudomonas* bacteremia in a 2-year-old sterilized by bacteriophage therapy. J. Pediatric Infect. Dis. Soc. 7, 253–256. 10.1093/jpids/pix056 28992111

[B58] Durante-Mangoni E. Signoriello G. Andini R. Mattei A. De Cristoforo M. Murino P.. (2013). Colistin and rifampicin compared with colistin alone for the treatment of serious infections due to extensively drug-resistant *Acinetobacter baumannii*: a multicenter, randomized clinical trial. Clin. Infect. Dis. 57, 349–358. 10.1093/cid/cit253 23616495

[B59] Dutta D. Ozkan J. Willcox M. D. P. (2014). Biocompatibility of antimicrobial melimine lenses. Optom. Vis. Sci. 91, 570–581. 10.1097/OPX.0000000000000232 24759327

[B60] Dvořáčková M. Růžička F. Benešík M. Pantůček R. Dvoráková-Heroldová M. (2018). Antimicrobial effect of commercial phage preparation Stafal® on biofilm and planktonic forms of methicillin-resistant *Staphylococcus aureus*. Folia Microbiol. 64, 121–126. 10.1007/s12223-018-0622-3 29923129

[B61] El-Azizi M. Khardori N. (2016). Efficacy of ultraviolet C light at sublethal dose in combination with antistaphylococcal antibiotics to disinfect catheter biofilms of methicillin-susceptible and methicillin-resistant *Staphylococcus aureus* and *Staphylococcus epidermidis in vitro*. Infect. Drug Resist. Volume 9, 181–189. 10.2147/IDR.S109343 27578990 PMC4998029

[B62] El-Shibiny A. El-Sahhar S. (2017). Bacteriophages: the possible solution to treat infections caused by pathogenic bacteria. Can. J. Microbiol. 63, 865–879. 10.1139/cjm-2017-0030 28863269

[B63] Fadlallah A. Chelala E. Legeais J. M. (2015). Corneal infection therapy with topical bacteriophage administration. Open Ophthalmol. J. 9, 167–168. 10.2174/1874364101509010167 26862360 PMC4740968

[B64] Fallah K. N. (2018). The Effects of Combination Antibiotic Therapy on Methicillin-Resistant Staphylococcus aureus. Doctoral dissertation.

[B65] Fila G. Kawiak A. Grinholc M. S. (2017). Blue light treatment of *Pseudomonas aeruginosa*: strong bactericidal activity, synergism with antibiotics and inactivation of virulence factors. Virulence 8, 938–958. 10.1080/21505594.2016.1250995 27763824 PMC5626244

[B66] Fish R. Kutter E. Wheat G. Blasdel B. Kutateladze M. Kuhl S. (2016). Bacteriophage treatment of intransigent diabetic toe ulcers: a case series. J. Wound Care 25, S27–S33. 10.12968/jowc.2016.25.Sup7.S2726949862

[B67] Founou R. C. Founou L. L. Essack S. Y. (2017). Clinical and economic impact of antibiotic resistance in developing countries: a systematic review and meta-analysis. PLoS ONE 12:e0189621. 10.1371/journal.pone.0189621 29267306 PMC5739407

[B68] Gaglione R. Dell'Olmo E. Bosso A. Chino M. Pane K. Ascione F.. (2017). Novel human bioactive peptides identified in Apolipoprotein B: evaluation of their therapeutic potential. Biochem. Pharmacol. 130, 34–50. 10.1016/j.bcp.2017.01.009 28131846

[B69] Gandt A. Griffith E. C. Lister I. M. Billings L. L. Han A. Tangallapally R. (2018). *In vivo* and *in vitro* effects of a ClpP-activating antibiotic against vancomycin-resistant *Enterococci*. Antimicrob. Agents Chemother. 62, e00424–e00418. 10.1128/AAC.00424-18 29784838 PMC6105829

[B70] Ghosh S. Patil S. Ahire M. Kitture R. Kale S. Pardesi K.. (2012). Synthesis of silver nanoparticles using *Dioscorea bulbifera* tuber extract and evaluation of its synergistic potential in combination with antimicrobial agents. Int. J. Nanomed. 7, 483–96. 10.2147/IJN.S24793 22334779 PMC3273981

[B71] Gill J. S. Arora S. Khanna S. P. Kumar K. H. (2016). Prevalence of multidrug-resistant, extensively drug-resistant, and pandrug-resistant *Pseudomonas aeruginosa* from a tertiary level intensive care unit. J. Glob. Infect. Dis. 8, 155–159. 10.4103/0974-777X.192962 27942195 PMC5126754

[B72] Goic-Barisic I. Seruga Music M. Kovacic A. Tonkic M. Hrenovic J. (2017). Pan drug-resistant environmental isolate of *Acinetobacter baumannii* from croatia. Microb. Drug Resist. 23, 494–496. 10.1089/mdr.2016.0229 27792476

[B73] Golińska P. Wypij M. Rathod D. Tikar S. Dahm H. Rai M. (2016). Synthesis of silver nanoparticles from two acidophilic strains of *Pilimelia columellifera* subsp. pallida and their antibacterial activities. J. Basic Microbiol. 56, 541–556. 10.1002/jobm.201500516 27151174

[B74] Gomes B. Augusto M. T. Felício M. R. Hollmann A. Franco O. L. Gonçalves S.. (2018). Designing improved active peptides for therapeutic approaches against infectious diseases. Biotechnol. Adv. 36, 415–429. 10.1016/j.biotechadv.2018.01.004 29330093

[B75] Gong P. Cheng M. Li X. Jiang H. Yu C. Kahaer N.. (2016). Characterization of *Enterococcus faecium* bacteriophage IME-EFm5 and its endolysin LysEFm5. Virology 492, 11–20. 10.1016/j.virol.2016.02.006 26896930

[B76] González-Bello C. (2017). Antibiotic adjuvants – a strategy to unlock bacterial resistance to antibiotics. Bioorg. Med. Chem. Lett. 27, 4221–4228. 10.1016/j.bmcl.2017.08.027 28827113

[B77] Górski A. Jończyk-Matysiak E. Łusiak-Szelachowska M. Miȩdzybrodzki R. Weber-Dąbrowska B. Borysowski J. (2017). The potential of phage therapy in sepsis. Front. Immunol. 8:1783. 10.3389/fimmu.2017.01783 29312312 PMC5732260

[B78] Göttig S. Gruber T. M. Higgins P. G. Wachsmuth M. Seifert H. Kempf V. A. J. (2014). Detection of pan drug-resistant *Acinetobacter baumannii* in Germany. J. Antimicrob. Chemother. 69, 2578–2579. 10.1093/jac/dku170 24833751

[B79] Greimel F. Scheuerer C. Gessner A. Simon M. Kalteis T. Grifka J.. (2017). Efficacy of antibiotic treatment of implant-associated *Staphylococcus aureus* infections with moxifloxacin, flucloxacillin, rifampin, and combination therapy: an animal study. Drug Des. Devel. Ther. 11, 1729–1736. 10.2147/DDDT.S138888 28652709 PMC5476658

[B80] Habash M. B. Goodyear M. C. Park A. J. Surette M. D. Vis E. C. Harris R. J.. (2017). Potentiation of tobramycin by silver nanoparticles against *Pseudomonas aeruginosa* Biofilms. Antimicrob. Agents Chemother. 61:e00415-17. 10.1128/AAC.00415-17 28848007 PMC5655055

[B81] Hall Snyder A. D. Werth B. J. Nonejuie P. McRoberts J. P. Pogliano J. Sakoulas G.. (2016). Fosfomycin enhances the activity of daptomycin against vancomycin-resistant *Enterococci* in an *in vitro* pharmacokinetic-pharmacodynamic model. Antimicrob. Agents Chemother. 60, 5716–5723. 10.1128/AAC.00687-16 27431211 PMC5038233

[B82] Halstead F. D. Thwaite J. E. Burt R. Laws T. R. Raguse M. Moeller R.. (2016). Antibacterial activity of blue light against nosocomial wound pathogens growing planktonically and as mature biofilms. Appl. Environ. Microbiol. 82, 4006–4016. 10.1128/AEM.00756-16 27129967 PMC4907187

[B83] Hemeg H. (2017). Nanomaterials for alternative antibacterial therapy. Int. J. Nanomed. Vol. 12, 8211–8225. 10.2147/IJN.S132163 29184409 PMC5689025

[B84] Hu X. Huang Y. Y. Wang Y. Wang X. Hamblin M. R. (2018). Antimicrobial photodynamic therapy to control clinically relevant biofilm infections. Front. Microbiol. 9, 1–24. 10.3389/fmicb.2018.01299 29997579 PMC6030385

[B85] Huang L. Wang M. Dai T. Sperandio F. F. Huang Y. Y. Xuan Y. (2014). Antimicrobial photodynamic therapy with decacationic monoadducts and bisadducts of [70]fullerene: *in vitro* and *in vivo* studies. Nanomedicine 9, 253–266. 10.2217/nnm.13.22 23738632 PMC3859801

[B86] Ibrahim M. E. Bilal N. E. Hamid M. E. (2012). Increased multi-drug resistant *Escherichia coli* from hospitals in Khartoum state, Sudan. Afr. Health Sci. 12, 368–375. 10.4314/ahs.v12i3.19 23382754 PMC3557680

[B87] Irani N. Basardeh E. Samiee F. Fateh A. Shooraj F. Rahimi A.. (2018). The inhibitory effect of the combination of two new peptides on biofilm formation by *Acinetobacter baumannii*. Microb. Pathog. 121, 310–317. 10.1016/j.micpath.2018.05.051 29859290

[B88] Jamal M. Andleeb S. Jalil F. Imran M. Nawaz M. A. Hussain T.. (2019). Isolation, characterization and efficacy of phage MJ2 against biofilm forming multi-drug resistant *Enterobacter cloacae*. Folia Microbiol. 64, 101–111. 10.1007/s12223-018-0636-x 30090964

[B89] Jault P. Leclerc T. Jennes S. Pirnay J. P. Que Y. A. Resch G.. (2019). Efficacy and tolerability of a cocktail of bacteriophages to treat burn wounds infected by *Pseudomonas aeruginosa* (PhagoBurn): a randomised, controlled, double-blind phase 1/2 trial. Lancet Infect. Dis. 19, 35–45. 10.1016/S1473-3099(18)30482-1 30292481

[B90] Jennes S. Merabishvili M. Soentjens P. Pang K. W. Rose T. Keersebilck E.. (2017). Use of bacteriophages in the treatment of colistin-only-sensitive *Pseudomonas aeruginosa* septicaemia in a patient with acute kidney injury-a case report. Crit. Care 21:129. 10.1186/s13054-017-1709-y 28583189 PMC5460490

[B91] Jo A. Ding T. Ahn J. (2016). Synergistic antimicrobial activity of bacteriophages and antibiotics against *Staphylococcus aureus*. Food Sci. Biotechnol. 25, 935–940. 10.1007/s10068-016-0153-0 30263357 PMC6049171

[B92] Jorgensen S. C. J. Rybak M. J. (2018). Meropenem and vaborbactam: stepping up the battle against carbapenem-resistant Enterobacteriaceae. Pharmacotherapy 38, 444–461. 10.1002/phar.2092 29427523

[B93] Kalan L. Wright G. D. (2011). Antibiotic adjuvants: multicomponent anti-infective strategies. Expert Rev. Mol. Med. 13:e5. 10.1017/S1462399410001766 21342612

[B94] Kasithevar M. Periakaruppan P. Muthupandian S. Mohan M. (2017). Antibacterial efficacy of silver nanoparticles against multi-drug resistant clinical isolates from post-surgical wound infections. Microb. Pathog. 107, 327–334. 10.1016/j.micpath.2017.04.013 28411059

[B95] Katayama B. Ozawa T. Morimoto K. Awazu K. Ito N. Honda N.. (2018). Enhanced sterilization and healing of cutaneous pseudomonas infection using 5-aminolevulinic acid as a photosensitizer with 410-nm LED light. J. Dermatol. Sci. 90, 323–331. 10.1016/j.jdermsci.2018.03.001 29534858

[B96] Kaur I. (2016). Novel strategies to combat antimicrobial resistance. J. Infect. Dis. Ther. 4:292 10.4172/2332-0877.100029

[B97] Kishor C. Mishra R. R. Saraf S. K. Kumar M. Srivastav A. K. Nath G. (2016). Phage therapy of staphylococcal chronic osteomyelitis in experimental animal model. Indian J. Med. Res. 143, 87–94. 10.4103/0971-5916.178615 26997019 PMC4822375

[B98] Kulengowski B. Rutter W. C. Campion J. J. Lee G. C. Feola D. J. Burgess D. S. (2018). Effect of increasing meropenem MIC on the killing activity of meropenem in combination with amikacin or polymyxin B against MBL- and KPC-producing *Enterobacter cloacae*. Diagn. Microbiol. Infect. Dis. 92, 262–266. 10.1016/j.diagmicrobio.2018.06.013 30098852

[B99] Kuo Y. L. Wang S. G. Wu C. Y. Lee K. C. Jao C. J. Chou S. H.. (2016). Functional gold nanoparticle-based antibacterial agents for nosocomial and antibiotic-resistant bacteria. Nanomedicine 11, 2497–2510. 10.2217/nnm-2016-0232 27622499

[B100] Latz S. Wahida A. Arif A. Häfner H. Hoß M. Ritter K.. (2016). Preliminary survey of local bacteriophages with lytic activity against multi-drug resistant bacteria. J. Basic Microbiol. 56, 1117–1123. 10.1002/jobm.201600108 27194637

[B101] LaVergne S. Hamilton T. Biswas B. Kumaraswamy M. Schooley R. T. Wooten D. (2018). Phage therapy for a multidrug-resistant *Acinetobacter baumannii* craniectomy site infection. Open Forum Infect. Dis. 5:ofy064. 10.1093/ofid/ofy064 29687015 PMC5905571

[B102] Lee N. Y. Lee C. C. Li C. W. Li M. C. Chen P. L. Chang C. M.. (2015). Cefepime therapy for monomicrobial *Enterobacter cloacae* bacteremia: unfavorable outcomes in patients infected by cefepime-susceptible dose-dependent isolates. Antimicrob. Agents Chemother. 59, 7558–7563. 10.1128/AAC.01477-15 26416853 PMC4649147

[B103] Leitner L. Sybesma W. Chanishvili N. Goderdzishvili M. Chkhotua A. Ujmajuridze A.. (2017). Bacteriophages for treating urinary tract infections in patients undergoing transurethral resection of the prostate: a randomized, placebo-controlled, double-blind clinical trial. BMC Urol. 17:90. 10.1186/s12894-017-0283-6 28950849 PMC5615798

[B104] Lewis K. (2007). Persister cells, dormancy and infectious disease. 5, 48–56. 10.1038/nrmicro1557 17143318

[B105] Liang D. Lu Z. Yang H. Gao J. Chen R. (2016). Novel asymmetric wettable AgNPs/Chitosan wound dressing: *in vitro* and *in vivo* evaluation. ACS Appl. Mater. Interfaces 8, 3958–3968. 10.1021/acsami.5b11160 26800283

[B106] Lin D. Koskella B. Lin H. C. (2017). Phage therapy: An alternative to antibiotics in the age of multi-drug resistance. World J. Gastrointest. Pharmacol. Ther. 8, 162–173. 10.4292/wjgpt.v8.i3.162 28828194 PMC5547374

[B107] Lin Q. Deslouches B. Montelaro R. C. Di Y. P. (2018). Prevention of ESKAPE pathogen biofilm formation by antimicrobial peptides WLBU2 and LL37. Int. J. Antimicrob. Agents. 52, 667–672. 10.1016/j.ijantimicag.2018.04.019 29753132 PMC6230315

[B108] Lin Y. Chang R. Y. K. Britton W. J. Morales S. Kutter E. Chan H.-K. (2018). Synergy of nebulized phage PEV20 and ciprofloxacin combination against *Pseudomonas aeruginosa*. Int. J. Pharm. 551, 158–165. 10.1016/j.ijpharm.2018.09.024 30223075 PMC6174101

[B109] Liu S. Long Q. Xu Y. Wang J. Xu Z. Wang L.. (2017). Assessment of antimicrobial and wound healing effects of Brevinin-2Ta against the bacterium *Klebsiella pneumoniae* in dermally-wounded rats. Oncotarget 8, 111369–111385. 10.18632/oncotarget.22797 29340060 PMC5762328

[B110] Long S. W. Olsen R. J. Mehta S. C. Palzkill T. Cernoch P. L. Perez K. K.. (2014). PBP2a mutations causing high-level Ceftaroline resistance in clinical methicillin-resistant *Staphylococcus aureus* isolates. Antimicrob. Agents Chemother. 58, 6668–6674. 10.1128/AAC.03622-14 25155594 PMC4249384

[B111] López-Cortés L. E. Cisneros J. M. Fernández-Cuenca F. Bou G. Tomás M. Garnacho-Montero J. (2014). Monotherapy versus combination therapy for sepsis due to multidrug-resistant *Acinetobacter baumannii*: analysis of a multicentre prospective cohort. J. Antimicrob. Chemother. 69, 3119–3126. 10.1093/jac/dku233 24970742

[B112] Lotha R. Shamprasad B. R. Sundaramoorthy N. S. Ganapathy R. Nagarajan S. Sivasubramanian A. (2018). Zero valent silver nanoparticles capped with capsaicinoids containing *Capsicum annuum* extract, exert potent anti-biofilm effect on food borne pathogen *Staphylococcus aureus* and curtail planktonic growth on a zebrafish infection model. Microb. Pathog. 124, 291–300. 10.1016/j.micpath.2018.08.053 30149130

[B113] Mahlapuu M. Håkansson J. Ringstad L. Björn C. (2016). Antimicrobial peptides: an emerging category of therapeutic agents. Front. Cell. Infect. Microbiol. 6:194. 10.3389/fcimb.2016.00194 28083516 PMC5186781

[B114] Mai B. Gao Y. Li M. Wang X. Zhang K. Liu Q.. (2017). Photodynamic antimicrobial chemotherapy for *Staphylococcus aureus* and multidrug-resistant bacterial burn infection *in vitro* and *in vivo*. Int. J. Nanomed. 12, 5915–5931. 10.2147/IJN.S138185 28860757 PMC5566361

[B115] Malik D. J. Sokolov I. J. Vinner G. K. Mancuso F. Cinquerrui S. Vladisavljevic G. T.. (2017). Formulation, stabilisation and encapsulation of bacteriophage for phage therapy. Adv. Colloid Interface Sci. 249, 100–133. 10.1016/j.cis.2017.05.014 28688779

[B116] Maliszewska I. Kałas W. Wysokiéska E. Tylus W. Pietrzyk N. Popko K.. (2018). Enhancement of photo-bactericidal effect of tetrasulfonated hydroxyaluminum phthalocyanine on *Pseudomonas aeruginosa*. Lasers Med. Sci. 33, 79–88. 10.1007/s10103-017-2337-0 28986706

[B117] Mandal S. M. Roy A. Ghosh A. K. Hazra T. K. Basak A. Franco O. L. (2014). Challenges and future prospects of antibiotic therapy: from peptides to phages utilization. Front. Pharmacol. 5:105. 10.3389/fphar.2014.00105 24860506 PMC4027024

[B118] Mann N. H. (2005). The third age of phage. PLoS Biol. 3:182. 10.1371/journal.pbio.0030182 15884981 PMC1110918

[B119] Manohar P. Nachimuthu R. Lopes B. S. (2018). The therapeutic potential of bacteriophages targeting gram-negative bacteria using *Galleria mellonella* infection model. BMC Microbiol. 18:97. 10.1186/s12866-018-1234-4 30170558 PMC6119258

[B120] Markoishvili K. Tsitlanadze G. Katsarava R. Morris J. G. Sulakvelidze A. (2002). A novel sustained-release matrix based on biodegradable poly(ester amide)s and impregnated with bacteriophages and an antibiotic shows promise in management of infected venous stasis ulcers and other poorly healing wounds. Int. J. Dermatol. 41, 453–458. 10.1046/j.1365-4362.2002.01451.x 12121566

[B121] Marsich E. Travan A. Donati I. Turco G. Kulkova J. Moritz N.. (2013). Biological responses of silver-coated thermosets: an *in vitro* and *in vivo* study. Acta Biomater. 9, 5088–5099. 10.1016/j.actbio.2012.10.002 23059413

[B122] Mcnair K. Bailey B. A. Edwards R. A. (2012). PHACTS, a computational approach to classifying the lifestyle of phages. Bioinformatics 28, 614–618. 10.1093/bioinformatics/bts014 22238260 PMC3289917

[B123] Mekkawy A. I. El-Mokhtar M. A. Nafady N. A. Yousef N. Hamad M. A. El-Shanawany S. M.. (2017). *In vitro* and *in vivo* evaluation of biologically synthesized silver nanoparticles for topical applications: effect of surface coating and loading into hydrogels. Int. J. Nanomedicine 12, 759–777. 10.2147/IJN.S124294 28176951 PMC5271388

[B124] Miller C. N. Newall N. Kapp S. E. Lewin G. Karimi L. Carville K.. (2010). A randomized-controlled trial comparing cadexomer iodine and nanocrystalline silver on the healing of leg ulcers. Wound Repair Regen. 18, 359–367. 10.1111/j.1524-475X.2010.00603.x 20636550

[B125] Mohanty S. Mishra S. Jena P. Jacob B. Sarkar B. Sonawane A. (2012). An investigation on the antibacterial, cytotoxic, and antibiofilm efficacy of starch-stabilized silver nanoparticles. Nanomedicine 8, 916–924. 10.1016/j.nano.2011.11.007 22115597

[B126] Mohapatra D. P. Debata N. K. Singh S. K. (2018). Extensively drug-resistant and pan-drug resistant Gram-negative bacteria in a tertiary-care hospital in Eastern India: A 4 year retrospective study. J. Glob. Antimicrob. Resist. 15, 246–249. 10.1016/j.jgar.2018.08.010 30144638

[B127] Möhler J. S. Sim W. Blaskovich M. A. T. Cooper M. A. Ziora Z. M. (2018). Silver bullets: a new lustre on an old antimicrobial agent. Biotechnol. Adv. 36, 1391–1411. 10.1016/j.biotechadv.2018.05.004 29847770

[B128] Monogue M. L. Giovagnoli S. Bissantz C. Zampaloni C. Nicolau D. P. (2018a). *In vivo* efficacy of meropenem, with a novel non-β-lactam β-lactamase inhibitor, nacubactam, against gram-negative organisms exhibiting various resistance mechanisms in a murine complicated urinary tract infection model. Antimicrob. Agents Chemother. 62:e02596-17. 10.1128/AAC.02596-17 30012751 PMC6125527

[B129] Monogue M. L. Sakoulas G. Nizet V. Nicolau D. P. (2018b). Humanized exposures of a β-lactam-β-lactamase inhibitor, tazobactam, versus non-β-lactam-β-lactamase inhibitor, avibactam, with or without colistin, against *Acinetobacter baumannii* in murine thigh and lung infection models. Pharmacology 101, 255–261. 10.1159/000486445 29533955

[B130] Moon S. H. Zhang X. Zheng G. Meeker D. G. Smeltzer M. S. Huang E. (2017). Novel linear lipopeptide paenipeptins with potential for eradicating biofilms and sensitizing gram-negative bacteria to rifampicin and clarithromycin. J. Med. Chem. 60, 9630–9640. 10.1021/acs.jmedchem.7b01064 29136469 PMC12124638

[B131] Morimoto K. Ozawa T. Awazu K. Ito N. Honda N. Matsumoto S.. (2014). Photodynamic therapy using systemic administration of 5-aminolevulinic acid and a 410-nm wavelength light-emitting diode for methicillin-resistant *Staphylococcus aureus*-infected ulcers in mice. PLoS ONE 9:e105173. 10.1371/journal.pone.0105173 25140800 PMC4139315

[B132] Munger M. A. Radwanski P. Hadlock G. C. Stoddard G. Shaaban A. Falconer J.. (2014). *In vivo* human time-exposure study of orally dosed commercial silver nanoparticles. Nanomed. Nanotechnol. Biol. Med. 10, 1–9. 10.1016/j.nano.2013.06.010 23811290 PMC3877176

[B133] Nakonieczna J. Wolnikowska K. Ogonowska P. Neubauer D. Bernat A. Kamysz W. (2018). Rose bengal-mediated photoinactivation of multidrug resistant *Pseudomonas aeruginosa* is enhanced in the presence of antimicrobial peptides. Front. Microbiol. 9:1949. 10.3389/fmicb.2018.01949 30177928 PMC6110182

[B134] Navidinia M. (2016). The clinical importance of emerging ESKAPE pathogens in nosocomial infections. J. Paramed. Sci. 7, 2008–4978. 10.22037/jps.v7i3.12584

[B135] Nigo M. Diaz L. Carvajal L. P. Tran T. T. Rios R. Panesso D.. (2017). Ceftaroline-resistant, daptomycin-tolerant, and heterogeneous vancomycin-intermediate methicillin-resistant *Staphylococcus aureus* causing infective endocarditis. Antimicrob. Agents Chemother. 61:e01235-16. 10.1128/AAC.01235-16 28232309 PMC5328541

[B136] Nour El Din S. El-Tayeb T. A. Abou-Aisha K. El-Azizi M. (2016). *In vitro* and *in vivo* antimicrobial activity of combined therapy of silver nanoparticles and visible blue light against *Pseudomonas aeruginosa*. Int. J. Nanomed. 11, 1749–1758. 10.2147/IJN.S102398 27175075 PMC4854264

[B137] Nowak J. Zander E. Stefanik D. Higgins P. G. Roca I. Vila J.. (2017). High incidence of pandrug-resistant *Acinetobacter baumannii* isolates collected from patients with ventilator-associated pneumonia in Greece, Italy and Spain as part of the magicbullet clinical trial. J. Antimicrob. Chemother. 72, 3277–3282. 10.1093/jac/dkx322 28961773 PMC5890771

[B138] Ostorhazi E. Hoffmann R. Herth N. Wade J. D. Kraus C. N. Otvos L. (2018). Advantage of a narrow spectrum host defense (Antimicrobial) peptide over a broad spectrum analog in preclinical drug development. Front. Chem. 6:359. 10.3389/fchem.2018.00359 30186829 PMC6111444

[B139] Otvos L. Ostorhazi E. Szabo D. Zumbrun S. D. Miller L. L. Halasohoris S. A.. (2018). Synergy between proline-rich antimicrobial peptides and small molecule antibiotics against selected gram-negative pathogens *in vitro* and *in vivo*. Front. Chem. 6:309. 10.3389/fchem.2018.00309 30155456 PMC6102830

[B140] Palavutitotai N. Jitmuang A. Tongsai S. Kiratisin P. Angkasekwinai N. (2018). Epidemiology and risk factors of extensively drug-resistant *Pseudomonas aeruginosa* infections. PLoS ONE 13:e0193431. 10.1371/journal.pone.0193431 29470531 PMC5823452

[B141] Pallavali R. R. Degati V. L. Lomada D. Reddy M. C. Durbaka V. R. P. (2017). Isolation and *in vitro* evaluation of bacteriophages against MDR-bacterial isolates from septic wound infections. PLoS ONE 12:e0179245. 10.1371/journal.pone.0179245 28719657 PMC5515400

[B142] Panáček A. Smékalová M. Kilianová M. Prucek R. Bogdanová K. Večeřová R.. (2015). Strong and nonspecific synergistic antibacterial efficiency of antibiotics combined with silver nanoparticles at very low concentrations showing no cytotoxic effect. Molecules 21:26. 10.3390/molecules21010026 26729075 PMC6273824

[B143] Pendleton J. N. Gorman S. P. Gilmore B. F. (2013). Clinical relevance of the ESKAPE pathogens. Expert Rev. Anti. Infect. Ther. 11, 297–308. 10.1586/eri.13.12 23458769

[B144] Peng Y. Song C. Yang C. Guo Q. Yao M. (2017). Low molecular weight chitosan-coated silver nanoparticles are effective for the treatment of MRSA-infected wounds. Int. J. Nanomed. 12, 295–304. 10.2147/IJN.S122357 28115847 PMC5221798

[B145] Pfalzgraff A. Brandenburg K. Weindl G. (2018). Antimicrobial peptides and their therapeutic potential for bacterial skin infections and wounds. Front. Pharmacol. 9:281. 10.3389/fphar.2018.00281 29643807 PMC5882822

[B146] Philipson C. Voegtly L. Lueder M. Long K. Rice G. Frey K.. (2018). Characterizing phage genomes for therapeutic applications. Viruses 10:188. 10.3390/v10040188 29642590 PMC5923482

[B147] Pirnay J. P. Verbeken G. Ceyssens P. J. Huys I. De Vos D. Ameloot C.. (2018). The magistral phage. Viruses 10:64. 10.3390/v10020064 29415431 PMC5850371

[B148] Pletzer D. Mansour S. C. Hancock R. E. W. (2018). Synergy between conventional antibiotics and anti-biofilm peptides in a murine, sub-cutaneous abscess model caused by recalcitrant ESKAPE pathogens. PLoS Pathog. 14:e1007084. 10.1371/journal.ppat.1007084 29928049 PMC6013096

[B149] Pompilio A. Geminiani C. Bosco D. Rana R. Aceto A. Bucciarelli T.. (2018). Electrochemically synthesized silver nanoparticles are active against planktonic and biofilm cells of *Pseudomonas aeruginosa* and other cystic fibrosis-associated bacterial pathogens. Front. Microbiol. 9:1349. 10.3389/fmicb.2018.01349 30026732 PMC6041389

[B150] Prakash V. Mishra P. Premi H. Walia A. Dhawan S. Kumar A. (2014). Increasing incidence of multidrug resistant *Pseudomonas aeruginosa* in inpatients of a tertiary care hospital. Int. J. Res. Med. Sci. 2:1302 10.5455/2320-6012.ijrms20141111

[B151] Qayyum S. Oves M. Khan A. U. (2017). Obliteration of bacterial growth and biofilm through ROS generation by facilely synthesized green silver nanoparticles. PLoS ONE 12:0181363. 10.1371/journal.pone.0181363 28771501 PMC5542591

[B152] Radulescu M. Andronescu E. Dolete G. Popescu R. C. Fufă O. Chifiriuc M. C.. (2016). Silver nanocoatings for reducing the exogenous microbial colonization of wound dressings. Materials 9:345. 10.3390/ma9050345 28773468 PMC5503056

[B153] Rath G. Hussain T. Chauhan G. Garg T. Goyal A. K. (2016). Collagen nanofiber containing silver nanoparticles for improved wound-healing applications. J. Drug Target. 24, 520–529. 10.3109/1061186X.2015.1095922 26487102

[B154] Regeimbal J. M. Jacobs A. C. Corey B. W. Henry M. S. Thompson M. G. Pavlicek R. L.. (2016). Personalized therapeutic cocktail of wild environmental phages rescues mice from *Acinetobacter baumannii* wound infections. Antimicrob. Agents Chemother. 60, 5806–5816. 10.1128/AAC.02877-15 27431214 PMC5038255

[B155] Reinhardt A. Neundorf I. (2016). Design and application of antimicrobial peptide conjugates. Int. J. Mol. Sci. 17:E701. 10.3390/ijms17050701 27187357 PMC4881524

[B156] Rice L. B. (2008). Federal funding for the study of antimicrobial resistance in nosocomial pathogens: no ESKAPE. J. Infect. Dis. 197, 1079–1081. 10.1086/533452 18419525

[B157] Rineh A. Bremner J. B. Hamblin M. R. Ball A. R. Tegos G. P. Kelso M. J. (2018). Attaching NorA efflux pump inhibitors to methylene blue enhances antimicrobial photodynamic inactivation of *Escherichia coli* and *Acinetobacter baumannii in vitro* and *in vivo*. Bioorg. Med. Chem. Lett. 28, 2736–2740. 10.1016/j.bmcl.2018.02.041 29519734 PMC6108959

[B158] Rineh A. Dolla N. K. Ball A. R. Magana M. Bremner J. B. Hamblin M. R.. (2017). Attaching the NorA efflux pump inhibitor INF55 to methylene blue enhances antimicrobial photodynamic inactivation of methicillin-resistant *Staphylococcus aureus in vitro* and *in vivo*. ACS Infect. Dis. 3, 756–766. 10.1021/acsinfecdis.7b00095 28799332 PMC6225778

[B159] Rios A. C. Moutinho C. G. Pinto F. C. Del Fiol F. S. Jozala A. Chaud M. V.. (2016). Alternatives to overcoming bacterial resistances: state-of-the-art. Microbiol. Res. 191, 51–80. 10.1016/j.micres.2016.04.008 27524653

[B160] Rishi N. P. Vashist T. Sharma A. Kaur A. Kaur A. Kaur N.. (2018). Efficacy of designer K11 antimicrobial peptide (a hybrid of melittin, cecropin A1 and magainin 2) against *Acinetobacter baumannii* infected wounds. Pathog. Dis. 76:fty072. 10.1093/femspd/fty072 30184071

[B161] Rohde C. Resch G. Pirnay J. P. Blasdel B. Debarbieux L. Gelman D.. (2018). Expert opinion on three phage therapy related topics: bacterial phage resistance, phage training and prophages in bacterial production strains. Viruses 10:178. 10.3390/v10040178 29621199 PMC5923472

[B162] Ronqui M. R. de Aguiar Coletti T. M. S. F. de Freitas L. M. Miranda E. T. Fontana C. R. (2016). Synergistic antimicrobial effect of photodynamic therapy and ciprofloxacin. J. Photochem. Photobiol. B Biol. 158, 122–129. 10.1016/j.jphotobiol.2016.02.036 26971277

[B163] Rubino C. M. Bhavnani S. M. Loutit J. S. Lohse B. Dudley M. N. Griffith D. C. (2018). Single-dose pharmacokinetics and safety of meropenem-vaborbactam in subjects with chronic renal impairment. Antimicrob. Agents Chemother. 62:e02103-17. 10.1128/AAC.02103-17 29311069 PMC5826113

[B164] Sangaonkar G. M. Pawar K. D. (2018). Garcinia indica mediated biogenic synthesis of silver nanoparticles with antibacterial and antioxidant activities. Colloids Surf. B. Biointerfaces 164, 210–217. 10.1016/j.colsurfb.2018.01.044 29413598

[B165] Santajit S. Indrawattana N. (2016). Mechanisms of antimicrobial resistance in ESKAPE pathogens. Biomed Res. Int. 2016:2475067. 10.1155/2016/2475067 27274985 PMC4871955

[B166] Santos A. L. Sodre C. L. Valle R. S. Silva B. A. Souza-Goncalves A. L. Sangenito L. S.. (2012). Antimicrobial action of chelating agents: repercussions on the microorganism development, virulence and pathogenesis. Curr. Med. Chem. 19, 2715–2737. 10.2174/092986712800609788 22455582

[B167] Schooley R. T. Biswas B. Gill J. J. Hernandez-Morales A. Lancaster J. Lessor L.. (2017). Development and use of personalized bacteriophage-based therapeutic cocktails to treat a patient with a disseminated resistant *Acinetobacter baumannii* Infection. Antimicrob. Agents Chemother. 61:e00954-17. 10.1128/AAC.00954-17 28807909 PMC5610518

[B168] Seo M. D. Won H. S. Kim J. H. Mishig-Ochir T. Lee B. J. Seo M. D.. (2012). Antimicrobial peptides for therapeutic applications: a review. Molecules 17, 12276–12286. 10.3390/molecules171012276 23079498 PMC6268056

[B169] Servick K. (2016). Beleaguered phage therapy trial presses on. Science 352, 1506–1506. 10.1126/science.352.6293.1506 27339963

[B170] Shalumon K. T. Sheu C. Chen C. H. Chen S. H. Jose G. Kuo C. Y.. (2018). Multi-functional electrospun antibacterial core-shell nanofibrous membranes for prolonged prevention of post-surgical tendon adhesion and inflammation. Acta Biomater. 72, 121–136. 10.1016/j.actbio.2018.03.044 29626695

[B171] Shivaswamy V. C. Kalasuramath S. B. Sadanand C. K. Basavaraju A. K. Ginnavaram V. Bille S.. (2015). Ability of bacteriophage in resolving wound infection caused by multidrug-resistant *Acinetobacter baumannii* in uncontrolled diabetic rats. Microb. Drug Resist. 21, 171–177. 10.1089/mdr.2014.0120 25411824

[B172] Siddiqi K. S. Husen A. Rao R. A. K. (2018). A review on biosynthesis of silver nanoparticles and their biocidal properties. J. Nanobiotechnol. 16:14. 10.1186/s12951-018-0334-5 29452593 PMC5815253

[B173] Singh R. Vora J. Nadhe S. B. Wadhwani S. A. Shedbalkar U. U. Chopade B. A. (2018). Antibacterial activities of bacteriagenic silver nanoparticles against nosocomial *Acinetobacter baumannii*. J. Nanosci. Nanotechnol. 18, 3806–3815. 10.1166/jnn.2018.15013 29442713

[B174] Singla S. Harjai K. Katare O. P. Chhibber S. (2016). Encapsulation of bacteriophage in liposome accentuates its entry in to macrophage and shields it from neutralizing antibodies. PLoS ONE 11:e0153777. 10.1371/journal.pone.0153777 27115154 PMC4846161

[B175] Skinner K. Sandoe J. A. T. Rajendran R. Ramage G. Lang S. (2017). Efficacy of rifampicin combination therapy for the treatment of enterococcal infections assessed *in vivo* using a *Galleria mellonella* infection model. Int. J. Antimicrob. Agents 49, 507–511. 10.1016/j.ijantimicag.2016.12.006 28235571

[B176] Soothill J. S. (1992). Treatment of experimental infections of mice with bacteriophages. J. Med. Microbiol. 37, 258–261. 10.1099/00222615-37-4-258 1404324

[B177] Stanley I. J. Kajumbula H. Bazira J. Kansiime C. Rwego I. B. Asiimwe B. B. (2018). Multidrug resistance among *Escherichia coli* and *Klebsiella pneumoniae* carried in the gut of out-patients from pastoralist communities of Kasese district, Uganda. PLoS ONE 13:e0200093. 10.1371/journal.pone.0200093 30016317 PMC6049918

[B178] Sueoka K. Chikama T. Latief M. A. Ko J. A. Kiuchi Y. Sakaguchi T.. (2018). Time-dependent antimicrobial effect of photodynamic therapy with TONS 504 on *Pseudomonas aeruginosa*. Lasers Med. Sci. 33, 1455–1460. 10.1007/s10103-018-2490-0 29589177

[B179] Sybesma W. Rohde C. Bardy P. Pirnay J. P. Cooper I. Caplin J.. (2018). Silk route to the acceptance and re-implementation of bacteriophage therapy—part II. Antibiotics 7:35. 10.3390/antibiotics7020035 29690620 PMC6023077

[B180] Tacconelli E. Carrara E. Savoldi A. Harbarth S. Mendelson M. Monnet D. L.. (2018). Discovery, research, and development of new antibiotics: the WHO priority list of antibiotic-resistant bacteria and tuberculosis. Lancet Infect. Dis. 18, 318–327. 10.1016/S1473-3099(17)30753-3 29276051

[B181] Tegos G. P. Hamblin M. R. (2006). Phenothiazinium antimicrobial photosensitizers are substrates of bacterial multidrug resistance pumps. Antimicrob. Agents Chemother. 50, 196–203. 10.1128/AAC.50.1.196-203.2006 16377686 PMC1346798

[B182] Tegos G. P. Masago K. Aziz F. Higginbotham A. Stermitz F. R. Hamblin M. R. (2008). Inhibitors of bacterial multidrug efflux pumps potentiate antimicrobial photoinactivation. Antimicrob. Agents Chemother. 52, 3202–3209. 10.1128/AAC.00006-08 18474586 PMC2533468

[B183] Téllez G. A. Zapata J. A. Toro L. J. Henao D. C. Bedoya J. P. Rivera J. D.. (2018). Identification, characterization, immunolocalization, and biological activity of lucilin peptide. Acta Trop. 185, 318–326. 10.1016/j.actatropica.2018.06.003 29890152

[B184] Tomb R. M. Maclean M. Coia J. E. MacGregor S. J. Anderson J. G. (2017). Assessment of the potential for resistance to antimicrobial violet-blue light in *Staphylococcus aureus*. Antimicrob. Resist. Infect. Control 6:100. 10.1186/s13756-017-0261-5 29046782 PMC5639585

[B185] Tomb R. M. White T. A. Coia J. E. Anderson J. G. MacGregor S. J. Maclean M. (2018). Review of the comparative susceptibility of microbial species to photoinactivation using 380–480 nm violet-blue light. Photochem. Photobiol. 94, 445–458. 10.1111/php.12883 29350751

[B186] Ujmajuridze A. Chanishvili N. Goderdzishvili M. Leitner L. Mehnert U. Chkhotua A.. (2018). Adapted bacteriophages for treating urinary tract infections. Front. Microbiol. 9:1832. 10.3389/fmicb.2018.01832 30131795 PMC6090023

[B187] Ullah A. Zhang Y. Iqbal Z. Zhang Y. Wang D. Chen J.. (2018). Household light source for potent photo-dynamic antimicrobial effect and wound healing in an infective animal model. Biomed. Opt. Express 9, 1006–1019. 10.1364/BOE.9.001006 29541500 PMC5846510

[B188] Vazquez-grande G. Kumar A. (2015). Optimizing antimicrobial therapy of sepsis and septic shock : focus on antibiotic combination therapy. Semin Respir Crit Care Med. 1, 154–166. 10.1055/s-0034-1398742 25643278

[B189] Verbelen J. Hoeksema H. Heyneman A. Pirayesh A. Monstrey S. (2014). Aquacel® Ag dressing versus Acticoat^TM^ dressing in partial thickness burns: a prospective, randomized, controlled study in 100 patients. Part 1: burn wound healing. Burns 40, 416–427. 10.1016/j.burns.2013.07.008 24045072

[B190] Vestergaard M. Paulander W. Marvig R. L. Clasen J. Jochumsen N. Molin S.. (2016). Antibiotic combination therapy can select for broad-spectrum multidrug resistance in *Pseudomonas aeruginosa*. Int. J. Antimicrob. Agents 47, 48–55. 10.1016/j.ijantimicag.2015.09.014 26597931

[B191] Viertel T. M. Ritter K. Horz H. P. (2014). Viruses versus bacteria-novel approaches to phage therapy as a tool against multidrug-resistant pathogens. J. Antimicrob. Chemother. 69, 2326–2336. 10.1093/jac/dku173 24872344

[B192] Vuotto C. Longo F. Pascolini C. Donelli G. Balice M. P. Libori M. F.. (2017). Biofilm formation and antibiotic resistance in *Klebsiella* pneumoniae urinary strains. J. Appl. Microbiol. 123, 1003–1018. 10.1111/jam.13533 28731269

[B193] Wan G. Ruan L. Yin Y. Yang T. Ge M. Cheng X. (2016). Effects of silver nanoparticles in combination with antibiotics on the resistant bacteria *Acinetobacter baumannii*. Int. J. Nanomed. Vol. 11, 3789–3800. 10.2147/IJN.S104166 27574420 PMC4990392

[B194] Wang G. Li X. Zasloff M. (2010). A database view of naturally occurring antimicrobial peptides: nomenclature, classification and amino acid sequence analysis, in Antimicrobial Peptides: Discovery, Design and Novel Therapeutic Strategies (Oxfordshire: CABI), 1–21. 10.1079/9781845936570.0001

[B195] Wang S. Yan C. Zhang X. Shi D. Chi L. Luo G.. (2018). Antimicrobial peptide modification enhances the gene delivery and bactericidal efficiency of gold nanoparticles for accelerating diabetic wound healing. Biomater. Sci. 6, 2757–2772. 10.1039/C8BM00807H 30187036

[B196] Wang Y. Harrington O. D. Wang Y. Murray C. K. Hamblin M. R. Dai T. (2017). *In vivo* investigation of antimicrobial blue light therapy for multidrug- resistant *Acinetobacter baumannii* burn infections using bioluminescence imaging. J. Vis. Exp. 122:e54997 10.3791/54997 PMC5523652 28518072

[B197] Wang Z. Zheng P. Ji W. Fu Q. Wang H. Yan Y.. (2016). SLPW: a virulent bacteriophage targeting methicillin-resistant *Staphylococcus aureus* in vitro and *in vivo*. Front. Microbiol. 7:934. 10.3389/fmicb.2016.00934 27379064 PMC4908117

[B198] Wen X. Zhang X. Szewczyk G. El-Hussein A. Huang Y.-Y. Sarna T.. (2017). Potassium iodide potentiates antimicrobial photodynamic inactivation mediated by rose bengal in *in vitro* and *in vivo* studies. Antimicrob. Agents Chemother. 61:e00467-17. 10.1128/AAC.00467-17 28438946 PMC5487662

[B199] Wittebole X. De Roock S. Opal S. M. (2014). A historical overview of bacteriophage therapy as an alternative to antibiotics for the treatment of bacterial pathogens. Virulence 5, 226–235. 10.4161/viru.25991 23973944 PMC3916379

[B200] Wozniak A. Grinholc M. (2018). Combined antimicrobial activity of photodynamic inactivation and antimicrobials-state of the art. Front. Microbiol. 9:930. 10.3389/fmicb.2018.00930 29867839 PMC5952179

[B201] Wypij M. Świecimska M. Czarnecka J. Dahm H. Rai M. Golinska P. (2018). Antimicrobial and cytotoxic activity of silver nanoparticles synthesized from two haloalkaliphilic actinobacterial strains alone and in combination with antibiotics. J. Appl. Microbiol. 124, 1411–1424. 10.1111/jam.13723 29427473

[B202] Xie J. Li Y. Li J. Yan Z. Wang D. Guo X.. (2018). Potent effects of amino acid scanned antimicrobial peptide Feleucin-K3 analogs against both multidrug-resistant strains and biofilms of *Pseudomonas aeruginosa*. Amino Acids 50, 1471–1483. 10.1007/s00726-018-2625-4 30136030

[B203] Yadav R. Bulitta J. B. Wang J. Nation R. L. Landersdorfer C. B. (2017). Evaluation of pharmacokinetic/ pharmacodynamic model-based optimized combination regimens against multidrug-resistant *Pseudomonas aeruginosa* in a murine thigh infection model by using humanized dosing schemes. Antimicrob. Agents Chemother. 61:e01268-17. 10.1128/AAC.01268-17 28993331 PMC5700304

[B204] Yang H. Chen G. Hu L. Liu Y. Cheng J. Ye Y.. (2018a). Enhanced efficacy of imipenem-colistin combination therapy against multiple-drug-resistant *Enterobacter cloacae: in vitro* activity and a *Galleria mellonella* model. J. Microbiol. Immunol. Infect. 51, 70–75. 10.1016/j.jmii.2016.01.003 26906264

[B205] Yang H. Xu J. Li W. Wang S. Li J. Yu J.. (2018b). *Staphylococcus aureus* virulence attenuation and immune clearance mediated by a phage lysin-derived protein. EMBO J. 37:e98045. 10.15252/embj.201798045 30037823 PMC6120661

[B206] Yang M. Y. Chang K. C. Chen L. Y. Wang P. C. Chou C. C. Wu Z. B.. (2018). Blue light irradiation triggers the antimicrobial potential of ZnO nanoparticles on drug-resistant *Acinetobacter baumannii*. J. Photochem. Photobiol. B. 180, 235–242. 10.1016/j.jphotobiol.2018.02.003 29475122

[B207] Yoshizumi A. Ishii Y. Livermore D. M. Woodford N. Kimura S. Saga T.. (2013). Efficacies of calcium-EDTA in combination with imipenem in a murine model of sepsis caused by *Escherichia coli* with NDM-1 β-lactamase. J. Infect. Chemother. 19, 992–995. 10.1007/s10156-012-0528-y 23233082

[B208] Youn Jun S. Jin Jang I. Yoon S. Jang K. Yu K. S. Youn Cho J. (2017). Pharmacokinetics and tolerance of the phage endolysin-based candidate drug SAL200 after a single intravenous administration among healthy volunteers. Antimicrob. Agents Chemother. 61:e02629-16. 10.1128/AAC.02629-16 28348152 PMC5444177

[B209] Zeng K. J. Doi Y. Patil S. Huang X. Tian G. B. (2016). Emergence of the plasmid-mediated mcr-1 gene in colistin-resistant *Enterobacter aerogenes* and *Enterobacter cloacae*. Antimicrob. Agents Chemother. 60, 3862–3863. 10.1128/AAC.00345-16 26976876 PMC4879368

[B210] Zhang J. Xu L. L. Gan D. Zhang X. (2018). *In vitro* study of bacteriophage AB3 endolysin LysAB3 activity against *Acinetobacter baumannii* biofilm and biofilm-bound *A*. baumannii. Clin. Lab. 64, 1021–1030. 10.7754/Clin.Lab.2018.180342 29945306

[B211] Zhang Y. Dai T. Wang M. Vecchio D. Chiang L. Y. Hamblin M. R. (2015). Potentiation of antimicrobial photodynamic inactivation mediated by a cationic fullerene by added iodide: *in vitro* and *in vivo* studies. Nanomedicine 10, 603–614. 10.2217/nnm.14.131 25723093 PMC4899971

[B212] Zhao Y. Lu Z. Dai X. Wei X. Yu Y. Chen X.. (2018). Glycomimetic-conjugated photosensitizer for specific *Pseudomonas aeruginosa* recognition and targeted photodynamic therapy. Bioconjug. Chem. 29, 3222–3230. 10.1021/acs.bioconjchem.8b00600 30152991

[B213] Zheng Z. Tharmalingam N. Liu Q. Jayamani E. Kim W. Fuchs B. B.. (2017). Synergistic efficacy of *Aedes aegypti* antimicrobial peptide cecropin A2 and tetracycline against *Pseudomonas aeruginosa*. Antimicrob. Agents Chemother. 61:e00686-17. 10.1128/AAC.00686-17 28483966 PMC5487646

[B214] Zhou W. Feng Y. Zong Z. (2018). Two new lytic bacteriophages of the myoviridae family against carbapenem-resistant *Acinetobacter baumannii*. Front. Microbiol. 9:850. 10.3389/fmicb.2018.00850 29760690 PMC5936750

[B215] Zowawi H. M. Forde B. M. Alfaresi M. Alzarouni A. Farahat Y. Chong T. M.. (2015). Stepwise evolution of pandrug-resistance in *Klebsiella pneumoniae*. Sci. Rep. 5:15082. 10.1038/srep15082 26478520 PMC4609946

